# UBR5 targets tumor suppressor CDC73 proteolytically to promote aggressive breast cancer

**DOI:** 10.1038/s41419-022-04914-6

**Published:** 2022-05-12

**Authors:** Gang Xiang, Shuxuan Wang, Ling Chen, Mei Song, Xiaoxu Song, Huan Wang, Pengbo Zhou, Xiaojing Ma, Jing Yu

**Affiliations:** 1 Joint International Research Laboratory of Metabolic and Developmental Sciences, Sheng Yushou Center of Cell Biology and Immunology, School of Life Sciences and Biotechnology, Shanghai Jiao Tong University, Shanghai, 200240 China; 2 Department of Microbiology and Immunology, Weill Cornell Medicine, New York, NY 10065 USA; 3 Department of Pathology and Laboratory Medicine, Weill Cornell Medicine, New York, NY 10065 USA

**Keywords:** Breast cancer, Ubiquitylation, Tumour-suppressor proteins

## Abstract

UBR5, a HECT-domain E3 ubiquitin ligase, is an attractive therapeutic target for aggressive breast cancers. Defining the substrates of UBR5 is crucial for scientific understanding and clinical intervention. Here, we demonstrate that CDC73, a component of the RNA polymerase II-associated factor 1 complex, is a key substrate that impedes UBR5’s profound tumorigenic and metastatic activities in triple-negative breast cancer (TNBC) via mechanisms of regulating the expression of β-catenin and E-cadherin, tumor cell apoptosis and CD8^+^ T cell infiltration. Expression of *CDC73* is also negatively associated with the progression of breast cancer patients. Moreover, we show that UBR5 destabilizes CDC73 by polyubiquitination at Lys^243^, Lys^247^, and Lys^257^ in a non-canonical manner that is dependent on the non-phosphorylation state of CDC73 at Ser^465^. CDC73 could serve as a molecular switch to modulate UBR5’s pro-tumor activities and may provide a potential approach to developing breast cancer therapeutic interventions.

## Introduction

In 2020, breast cancer surpassed lung cancer as the most commonly diagnosed cancer out of 36 cancers from 185 countries with an estimated 2.3 million new cases, and it is the fifth leading cause of cancer mortality worldwide [1]. TNBC represents about 15% of all breast cancer cases and is characterized by rapid progression and poorer prognosis [2]. Despite tremendous progress in the development of diagnostic and treatment strategies, the overall survival of patients with breast cancer is not satisfactory and breast cancer remains the leading cause of cancer-related deaths in women [3]. Hence, it is important to discover novel targets and more efficacious therapeutic strategies in this deadly disease.

Human UBR5 (ubiquitin protein ligase E3 component n-recognin 5), also known as EDD, was originally identified in a screen for progestin-regulated genes in breast cancer cells, and it is a highly conserved HECT-domain E3 ubiquitin ligase [4]. Overexpression of UBR5 has been demonstrated in several cancers, including breast [5, 6], ovarian [7], colorectal [8], pancreatic [9], and gastric cancers [10], and is closely associated with advanced clinical stage, distant metastasis, and shorter overall survival in patients. UBR5 has been implicated in the regulation of DNA damage response [11], cell cycle [12], metabolism [13], transcription [14, 15], and apoptosis [16, 17]. In addition, it is responsible for breast and ovarian cancers’ resistance to tamoxifen and cisplatin, respectively [18, 19]. Emerging evidence suggests that UBR5 is a key regulator of the ubiquitin-proteasome system (UPS) in cancer progression, such as CDK9 in melanoma [20], C/EBPα in pancreatic cancer [9], and MOAP-1 in ovarian cancer [21]. And the development of UBR5 as a drug target will likely depend on defining its enzymatic substrates in a cancer context [22].

In our previous studies in murine models, we have demonstrated that UBR5, functioning like an “oncogene”, plays a profound role in promoting breast and ovarian cancer growth and metastasis [5–7]. However, the key targets and molecular mechanisms in UBR5-mediated breast cancer aggression remain poorly defined. To begin to identify potential UBR5 targets, we performed mass spectrometric analysis of 4T1 WT vs. 4T1/*Ubr5*^*−/−*^ cells, which revealed a number of proteins of differential levels including parafibromin or CDC73 among the 30 up-regulated proteins in 4T1/*Ubr5*^−/−^ cells, implying that UBR5 has a direct or indirect role in inhibiting these proteins [6].

Here, we report that CDC73 is a crucial substrate of the E3 ubiquitin ligase of UBR5 and define the key amino acids for the stabilization of CDC73 via a non-canonical protein turnover mechanism. Clinical metadata analysis demonstrates a strong negative association of *CDC73* expression with relapse free survival of breast cancer patients. Moreover, we present evidence that CDC73 modulates UBR5’s tumorigenic properties by tumor cell-intrinsic mechanisms as well as those that affect the tumor microenvironment (TME).

## Results

### *CDC73* expression is decreased in human breast cancer tissues and is associated with poor prognosis

**Fig. 1:**
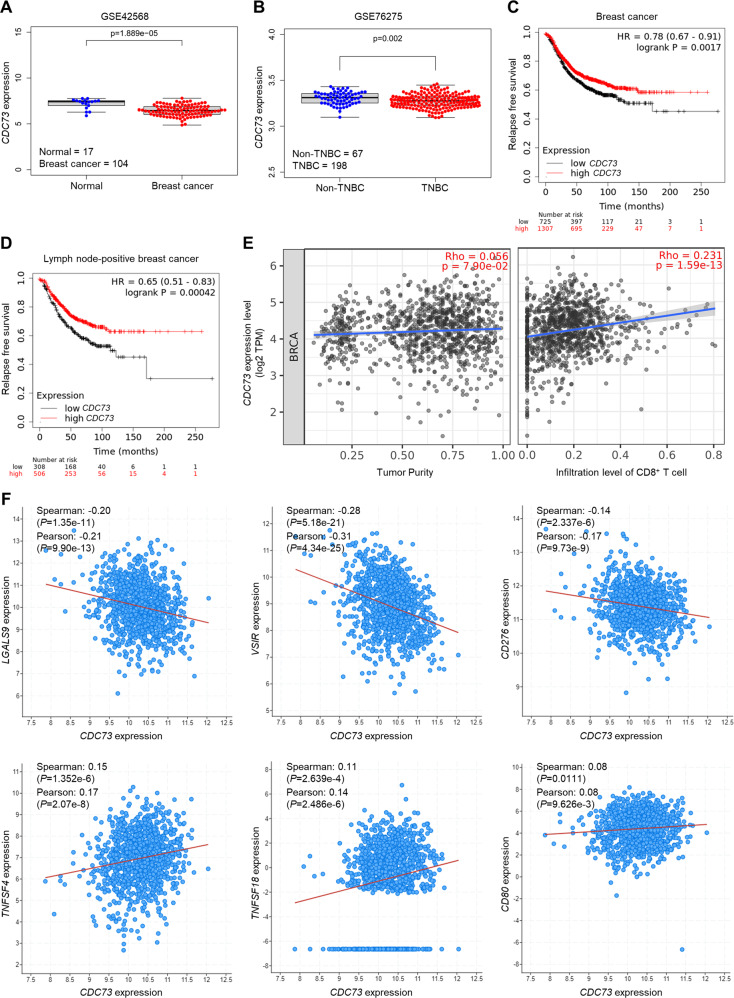
Expression of *CDC73* negatively correlates with human breast cancer progression. **A** The mRNA level of *CDC73* in patient normal breast tissue (*n* = 17) and breast cancer tissue (*n* = 104) from the subset GSE42568. **B** The mRNA level of *CDC73* in patient Non-TNBC tissue (*n* = 67) and TNBC tissue (*n* = 198) from the subset GSE76275. Correlations of *CDC73* expression with relapse free survival of breast cancer patients (*n* = 2032) (**C**) and lymph node-positive breast cancer patients (*n* = 814) (**D**) were analyzed by using the Kaplan–Meier plotter database. **E** Correlations of *CDC73* expression with the infiltration level of CD8^+^ T cells (right) in human breast cancer from TCGA BRCA cohort were analyzed by the TIMER2.0 database (*n* = 1100). **F** Correlations of the mRNA expression of CDC73 with the expression of immune response regulators *LGALS9*, *VSIR*, *CD276*, *TNFSF4*, *TNFSF18*, and *CD80* in human breast cancer from TCGA were analyzed by using the cBioPortal database (*n* = 1084).

We analyzed publicly available human patient-derived data from the Gene Expression Omnibus database (subset GSE42568 and GSE76275) to assess the expression level of *CDC73* in human breast cancer. The result shows that the mRNA level of *CDC73* is significantly lower in breast cancer than that in normal breast tissues (*p* = 1.889 × 10^−5^) (Fig. 1A) and lower still in TNBC than other breast cancer subtypes (*p* = 0.002) (Fig. 1B). In addition, low *CDC73* expression is significantly associated with poor relapse free survival of breast cancer patients (*p* = 0.0017) (Fig. 1C), particularly lymph node-positive breast cancer patients (*p* = 0.00042) (Fig. 1D). Interestingly, the expression level of *CDC73* is positively associated with the infiltration level of CD8^+^ T cells in human breast cancer (*p* = 1.59 × 10^−13^) (Fig. 1E). Furthermore, we investigated the correlation of the mRNA expression between *CDC73* and several crucial immune response regulators that are involved in regulating T cell activation in human breast cancer. The results show that *CDC73* expression is weakly but significantly negatively correlated with immune checkpoint molecules *LGALS9*, *VSIR*, and *CD276*. It also has a weak but significant positive correlation with costimulatory molecules *TNFSF4*, *TNFSF18*, and *CD80* expression (Fig. 1F). These data suggest that CDC73 may play an important role in limiting UBR5’s tumorigenic activities in a manner that involves immune cells in TME. Hence, we further explored the biological relationship between CDC73 and UBR5.

### The protein level of CDC73 is inhibited by UBR5 in breast cancer cells

**Fig. 2:**
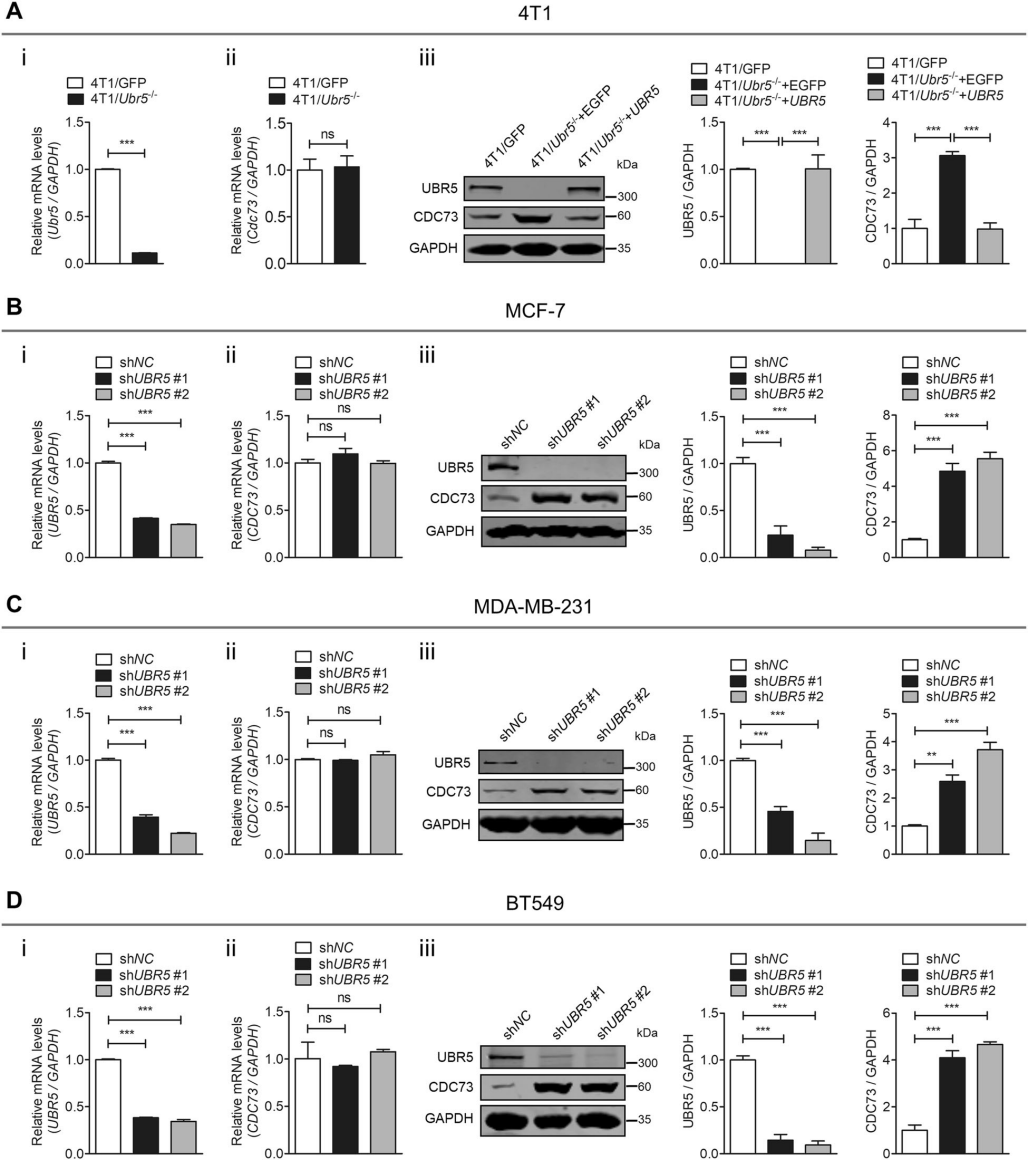
UBR5 inhibits the protein level of CDC73 in breast cancer cells. **A** The mRNA expression of *Ubr5* (i) and *Cdc73* (ii) was measured by qPCR, and their protein level (iii) was analyzed by western blotting in 4T1/GFP and 4T1/*Ubr5*^−/−^ cells. sh*NC* and *UBR5*-knocked down MCF-7 cells (**B**), MDA-MB-231 cells (**C**), and BT549 cells (**D**) were analyzed for the mRNA expression of *UBR5* (i) and *CDC73* (ii), and their protein level (iii) by qPCR and western blotting, respectively. Data were shown as mean ± SEM for triplicates; ns not significant; **p < 0.01; ***p < 0.001.

We next determined the regulatory mode of CDC73’s expression by UBR5 in vitro at both mRNA and protein levels in murine TNBC 4T1 cells. CRISPR-mediated knockout of *Ubr5* did not affect *Cdc73* mRNA expression, whereas it resulted in a strongly increased level of CDC73 protein (Fig. 2A). Ectopic expression of human *UBR5* in both WT 4T1 cells (Fig. S1A, B) and 4T1/*Ubr5*^−/−^ cells decreased the protein level of CDC73 (Fig. 2A). We then extended these analyses to human breast cancer cell lines, MCF-7 (Non-TNBC) (Fig. 2B), and TNBC cell lines MDA-MB-231 (Fig. 2C) and BT549 (Fig. 2D), all of which concurred with the finding in 4T1 cells. These data demonstrate that UBR5 controls CDC73 protein level in breast cancer and TNBC cells.

### CDC73 antagonizes UBR5’s tumorigenic and CD8^+^ T cell-suppressive activities

**Fig. 3:**
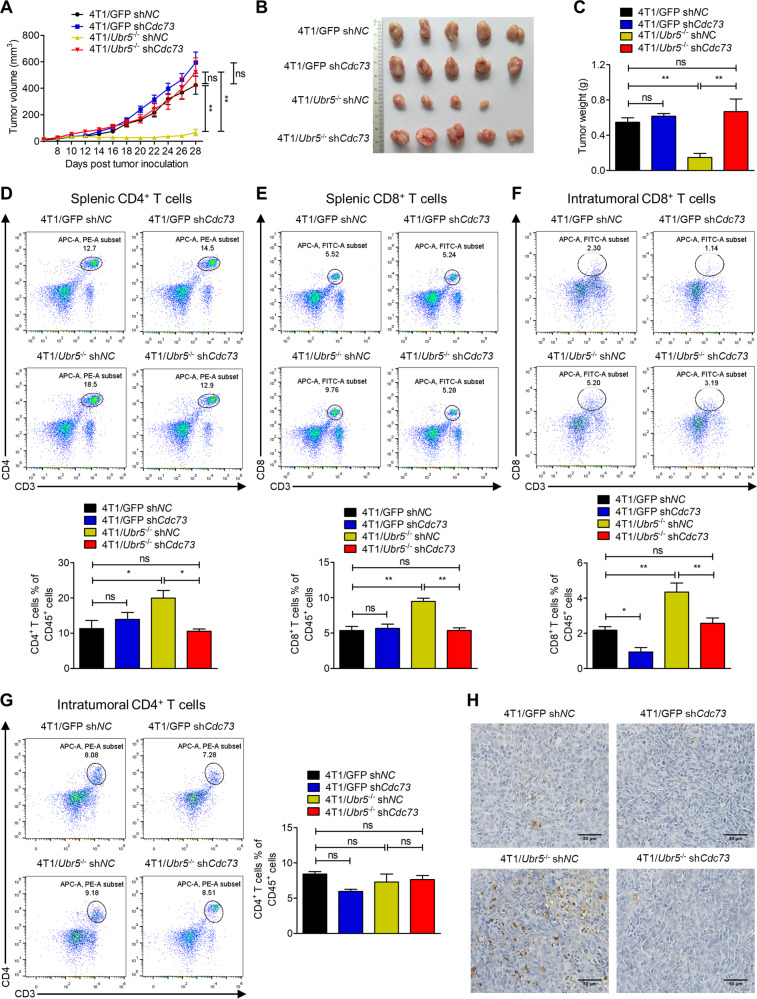
CDC73 antagonizes UBR5’s tumorigenic and immunoregulatory activities. **A** Tumor size was measured every 2 days in BALB/c mice injected with 5 × 10^5^ 4T1/GFP sh*NC*, 4T1/GFP sh*Cdc73*, 4T1/*Ubr5*^−/−^ sh*NC*, and 4T1/*Ubr5*^−/−^ sh*Cdc73* cells into the mammary fat pad, respectively, and the tumor-bearing mice were sacrificed on day 28 after injection (*n* = 5). Representative tumor images (**B**) were documented and tumor weight (**C**) was measured on day 28. Representative FACS images and quantification of splenic CD4^+^ T cells (**D**) and splenic CD8^+^ T cells (**E**), as well as intratumoral CD8^+^ T cells (**F**) and intratumoral CD4^+^ T cells (**G**) were analyzed on day 28 (*n* = 3). **H** Representative images of TUNEL staining of the tumor sections analyzed on day 28. Data were shown as mean ± SEM; ns not significant; *p < 0.05; **p < 0.01.

To evaluate the effects of CDC73 in UBR5-driven tumor growth, we further silenced the expression of *Cdc73* in 4T1/GFP and 4T1/*Ubr5*^−/−^ cells, as well as WT and *UBR5* knockdown cells of MCF-7, MDA-MB-231, and BT549 by *CDC73*-targeted shRNAs, and the knockdown efficiency was confirmed at both mRNA and protein levels (Fig. S2). Knocking down *CDC73* expression had little effects on cell proliferation and apoptosis of these cells in vitro (Fig. S3), whereas it almost completely restored tumor growth of 4T1/*Ubr5*^−/−^ cells in mice (Fig. 3A–C). Since we had previously observed significant increases in tumor-infiltrating T lymphocytes in mice carrying the 4T1/*Ubr5*^−/−^ tumor [6], it was of interest to note that both CD4^+^ and CD8^+^ T cells were markedly decreased in spleens in the 4T1/*Ubr5*^−/−^ sh*Cdc73* tumor-bearing mice (Fig. 3D, E), and intratumoral CD8^+^ T cells were also strongly inhibited (Fig. 3F), whereas CD4^+^ T cells were not affected (Fig. 3G). However, there were no obvious changes in natural killer cells and myeloid derived suppressor cells either in the spleen or in the tumor (Fig. S4). Moreover, compared to the WT tumor, *Ubr5*^−/−^ tumor exhibited increased apoptosis by TUNEL staining (Figs. 3H and S5A) and elevated levels of cleaved caspase-3 and cleaved PARP1 by immunohistochemistry (IHC) staining (Fig. S5B, C), respectively. However, the apoptosis was repressed in *Ubr5*^−/−^ sh*Cdc73* tumor (Figs. 3H and S5). Collectively, these results suggest that the tumor suppressor CDC73 is a key regulatory protein that can block UBR5’s activities on tumor growth and lymphocyte mobilization.

### CDC73 inhibits UBR5-driven tumor metastasis by down-regulating the expression of β-catenin and E-cadherin

**Fig. 4:**
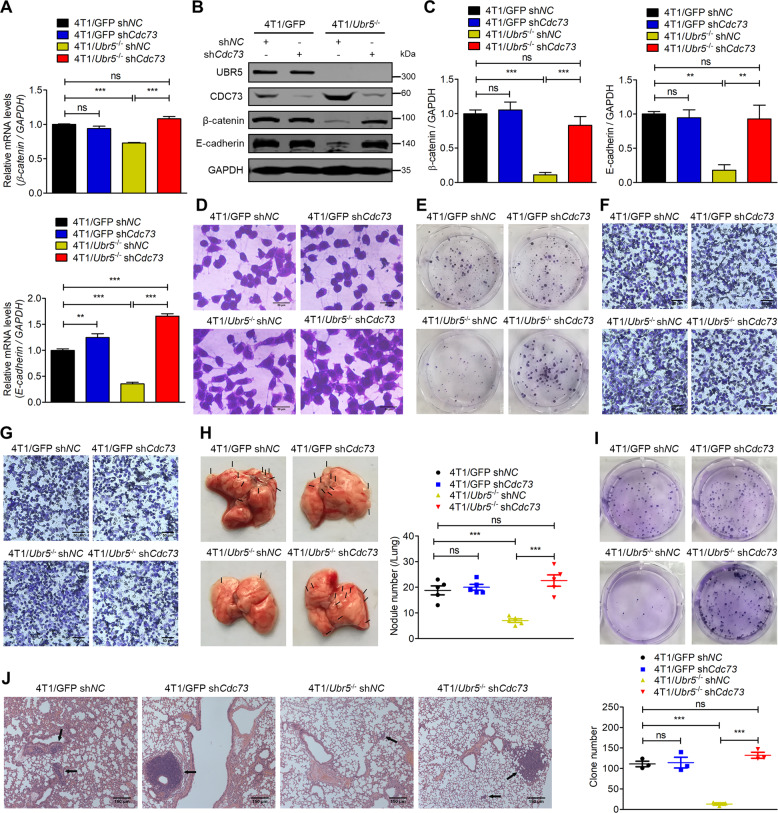
CDC73 inhibits UBR5’s metastatic activities. The mRNA expression (**A**), protein expression (**B**) and quantification (**C**) of β-catenin and E-cadherin were measured by qPCR and western blotting, respectively, in 4T1/GFP sh*NC*, 4T1/GFP sh*Cdc73*, 4T1/*Ubr5*^−/−^ sh*NC*, and 4T1/*Ubr5*^−/−^ sh*Cdc73* cells. **D** Representative images of the cell morphology analyzed for 4T1/GFP sh*NC*, 4T1/GFP sh*Cdc73*, 4T1/*Ubr5*^−/−^ sh*NC*, and 4T1/*Ubr5*^−/−^ sh*Cdc73* cells stained with 0.5% crystal violet. **E** Representative images of colonies of 4T1/GFP sh*NC*, 4T1/GFP sh*Cdc73*, 4T1/*Ubr5*^−/−^ sh*NC*, and 4T1/*Ubr5*^−/−^ sh*Cdc73* cells in colony formation assay in six-well plates (100 cells/well). Representative images of transwell migration (**F**) and invasion (**G**) were analyzed for 4T1/GFP sh*NC*, 4T1/GFP sh*Cdc73*, 4T1/*Ubr5*^−/−^ sh*NC*, and 4T1/*Ubr5*^−/−^ sh*Cdc73* cells, respectively. **H** Representative images of lungs in the tumor-bearing mice and the visible metastatic nodules on lung surfaces were counted. Female BALB/c mice were intravenously injected with 2 × 10^5^ 4T1/GFP sh*NC*, 4T1/GFP sh*Cdc73*, 4T1/*Ubr5*^−/−^ sh*NC*, and 4T1/*Ubr5*^−/−^ sh*Cdc73* cells, respectively. The tumor-bearing mice were sacrificed and the lungs were harvested on day 21 (*n* = 5). **I** Metastatic tumor cells of the lungs as described in (**H**) were visualized and quantitated by the 6-thioguanine clonogenicity assay (*n* = 3). **J** Representative images of H&E staining of lung sections. Data were presented as mean ± SEM; ns not significant; **p < 0.01; ***p < 0.001.

We previously reported that UBR5 drove 4T1 tumor metastasis primarily in a cell-autonomous manner and CRISPR-mediated deletion of *Ubr5* caused the reduction of the expression of several metastasis-promoting molecules including β-catenin and E-cadherin, as well as altering the cell morphology from an epithelial type to a mesenchymal type [5, 6]. Here, we observed that knocking down *Cdc73* expression in 4T1/*Ubr5*^−/−^ cells resulted in strongly increased expression of β-catenin and E-cadherin at both mRNA (Fig. 4A) and protein levels (Fig. 4B, C). Compared to the mesenchymal appearance of 4T1/*Ubr5*^−/−^ cells, the knocking down of *Cdc73* in these cells restored the cuboidal phenotype like that of the WT cells (Fig. 4D). Consistent with these molecular alterations, the clonogenicity, migratory and invasive capacities of *Ubr5*^−/−^ sh*Cdc73* cells fully reverted to those of the control and WT cells (Figs. 4E–G and S6). Moreover, 4T1/*Ubr5*^−/−^ sh*Cdc73* cells, when injected intravenously into mice, completely regained the WT capacity to metastasize to the lungs, as measured by the number of metastatic nodules in the lungs (Fig. 4H), tumor cell colonies grown in culture dishes in the presence of 6-thioguanine (Fig. 4I), and metastatic foci visualized by hematoxylin and eosin (H&E) staining (Fig. 4J). Taken together, these data demonstrate that CDC73 plays an essential role in constraining UBR5’s metastatic activities in a cell-intrinsic manner in addition to its cell extrinsic effects on CD8^+^ T cells.

### UBR5 directly interacts with CDC73 and controls the protein levels of PAF1C components

**Fig. 5:**
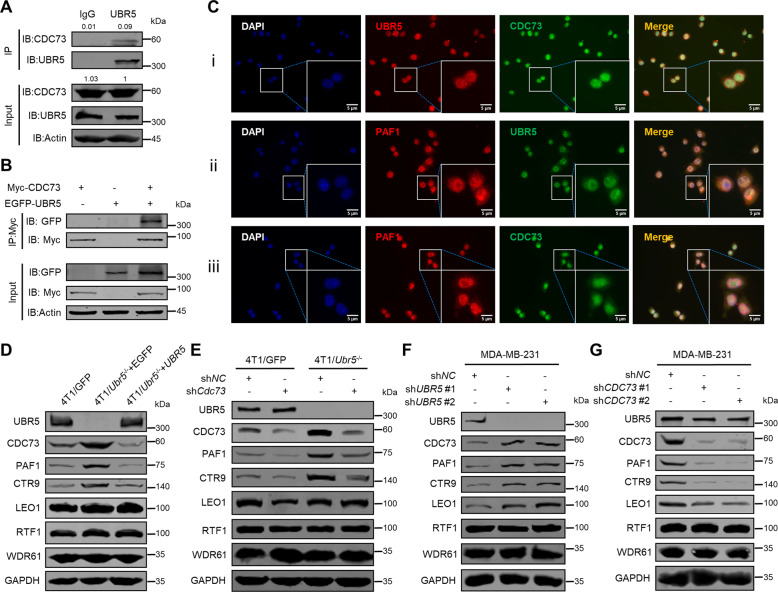
Interaction of UBR5 with CDC73/PAF1C. **A** Physical interaction of endogenous UBR5 with endogenous CDC73 was detected by co-IP with anti-UBR5 antibody in 4T1 cells which were treated with MG132 (10 μM) for 8 h before being collected for co-IP. **B** Physical interaction of exogenous UBR5 with exogenous CDC73 was monitored by co-IP with anti-Myc antibody in HEK293T cells transfected with empty vector, Myc-CDC73 or/and EGFP-UBR5 expressing plasmids for 40 h and then treated with MG132 (10 μM) for 8 h before being collected for co-IP. **C** The co-localization of endogenous UBR5 and CDC73 (i), UBR5 and PAF1 (ii), and CDC73 and PAF1 (iii) in 4T1 cells was monitored by immunofluorescence with a fluorescent microscope. The protein levels of UBR5 and the subunits of PAF1C including CDC73, PAF1, CTR9, LEO1, RTF1, and WDR61 were detected by western blotting in 4T1/GFP and 4T1/*Ubr5*^−/−^ cells reconstituted with EGFP or *UBR5* (**D**), 4T1/GFP sh*NC*, 4T1/GFP sh*Cdc73*, 4T1/*Ubr5*^−/−^ sh*NC*, and 4T1/*Ubr5*^−/−^ sh*Cdc73* cells (**E**), sh*NC* and *UBR5* knockdown MDA-MB-231 cells (**F**), and sh*NC* and *CDC73* knockdown MDA-MB-231 cells (**G**).

To further elucidate the biochemical basis of the regulatory relationship between UBR5 and CDC73, we conducted co-IP assays to assess the physical interaction of the endogenous proteins in 4T1 cells (Fig. 5A) and the interaction of the exogenously introduced, EGFP-tagged UBR5 and Myc-tagged CDC73 in HEK293T cells (Fig. 5B). The endogenous UBR5-CDC73 interaction in 4T1 cells was also corroborated by immunofluorescence microscopy (Fig. 5Ci). Moreover, the co-localization of UBR5, CDC73, and PAF1 was also observed mainly in the nucleus (Fig. 5Ci–iii). In addition to CDC73 and PAF1, we also analyzed the other four canonical components of the highly conserved RNA polymerase II-associated factor 1 complex (PAF1C) [23], which regulates transcriptional events and histone modifications in cell growth and survival [24–26]. Compared to 4T1/GFP cells, CTR9 protein level, like those of CDC73 and PAF1, was increased in 4T1/*Ubr5*^−/−^ cells and then decreased upon ectopic expression of human *UBR5*, whereas LEO1, RTF1, and WDR61 levels were not significantly altered (Fig. 5D). Similar to the findings in Hela cells in a previous study [27], silencing the expression of *Cdc73* in 4T1 cells by shRNA mainly reduced the protein levels of PAF1 and CTR9 while with little impact on other PAF1C subunits (Fig. 5E). These observations were also made in human TNBC MDA-MB-231 cells with respect to the role of UBR5 (Fig. 5F) and CDC73 (Fig. 5G) in regulating PAF1C components. These data demonstrate that UBR5 physically interacts with CDC73/PAF1C and the critical role of UBR5 in the control of the protein level of PAF1C, particularly CDC73, PAF1, CTR9, and LEO1, in TNBC cells.

### UBR5 destabilizes non-phosphorylated CDC73 at Ser^465^ by its E3 ligase activity via the proteasome

**Fig. 6:**
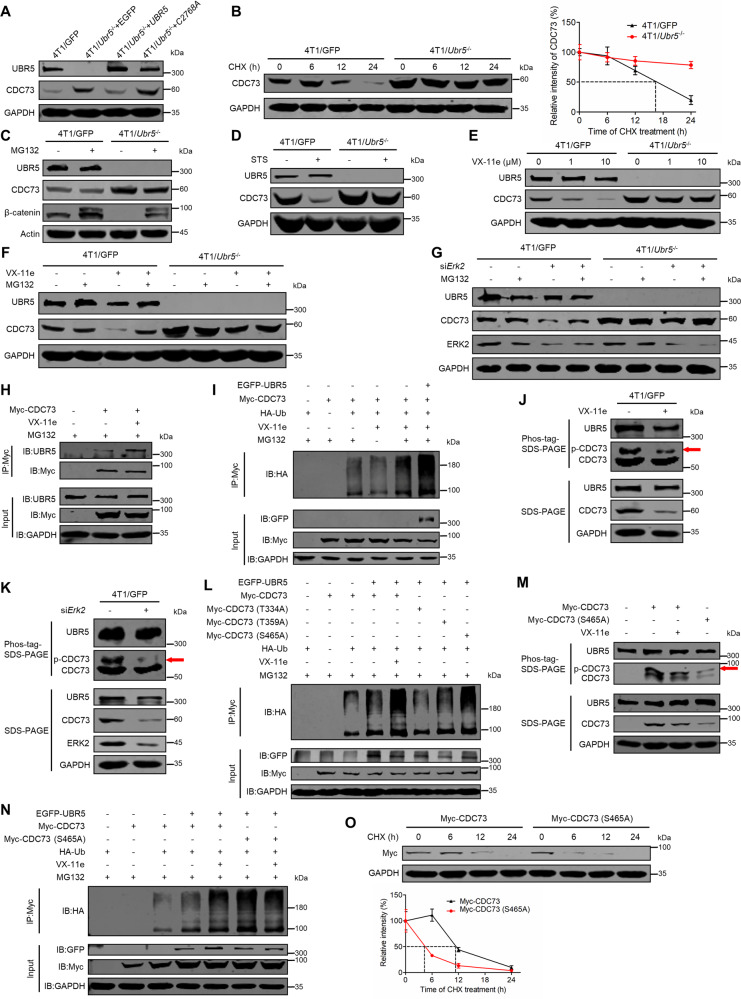
Degradation of non-phosphorylated CDC73 at Ser^465^ by UBR5-mediated UPS. **A** The protein levels of UBR5 and CDC73 in 4T1/GFP and 4T1/*Ubr5*^*−/−*^ cells reconstituted with EGFP, *UBR5*, or *UBR5 C2768A* mutant were evaluated by western blotting. **B** The turnover of CDC73 in 4T1/GFP and 4T1/*Ubr5*^*−/−*^ cells was measured by CHX treatment (50 μg/ml; 0, 6, 12 and 24 h) and then detected by western blotting and quantified by ImageJ software. The protein level of CDC73 was detected by western blotting in both 4T1/GFP and 4T1/*Ubr5*^*−/−*^ cells treated with vehicle, MG132 (10 μM, 8 h) (**C**), STS (0.1 μM, 6 h) (**D**), VX-11e (1 μM or 10 μM, 24 h) (**E**), and VX-11e (10 μM, 24 h) or/and MG132 (10 μM, 8 h) (**F**). **G** The protein level of CDC73 was detected by western blotting in both 4T1/GFP and 4T1/*Ubr5*^*−/−*^ cells transfected with control siRNA or si*Erk2* and treated with vehicle or MG132 (10 μM, 8 h). **H** Co-IP with anti-Myc antibody analyzed the physical interaction of endogenous UBR5 and exogenous CDC73 in HEK293T cells transfected with empty vector or Myc-CDC73 plasmids and treated with vehicle or VX-11e (10 μM, 24 h) in the presence of MG132 (10 μM, 8 h). **I** Ubiquitination assays analyzed the ubiquitination level of CDC73 modified by exogenous UBR5 in the presence of vehicle, VX-11e (10 μM, 24 h) or/and MG132 (10 μM, 8 h) under denaturing conditions in HEK293T cells transfected with the indicated plasmids. The phosphorylation levels of UBR5 and CDC73 were analyzed via the Phos-tag SDS-PAGE in 4T1 cells treated with vehicle or VX-11e (10 µM, 24 h) (**J**) and the control and *Erk2* silenced 4T1 cells (**K**). **L** Ubiquitination assays analyzed the ubiquitination level of three mutants (T334A, T359A and S465A) of CDC73 modified with exogenous UBR5. The phosphorylation (**M**) and ubiquitination (**N**) levels of WT and S465A mutant of CDC73 were detected in HEK293T cells treated with vehicle or VX-11e (10 μM, 24 h). **O** The turnover of WT and the S465A mutant of CDC73. HEK293T cells were transfected with Myc-CDC73 and Myc-CDC73 (S465A) plasmids for 48 h and treated with CHX as described in (**B**).

To further understand how CDC73 is regulated by UBR5 at the protein level, we analyzed CDC73 protein levels by western blotting, which showed increased CDC73 in 4T1/*Ubr5*^−/−^ cells when compared to 4T1/GFP cells (Fig. 6A). Reconstitution of 4T1/*Ubr5*^−/−^ cells with human *UBR5* but not its ubiquitin ligase-deficient mutant *C2768A* reduced CDC73 to the WT level (Fig. 6A), suggesting that CDC73 may be destabilized via UBR5’s E3 ubiquitin ligase activity. Moreover, the half-life of CDC73 protein was greatly extended in the absence of UBR5 in cells treated with the protein synthesis inhibitor cycloheximide (CHX) from 17 h to well above 24 h (Fig. 6B), further supporting the notion of the involvement of a protein degradation mechanism. Hence, we next investigated whether the degradation of CDC73 by UBR5 was through the classic UPS. However, treatment of 4T1/GFP cells with the proteasome inhibitor MG132 or bortezomib (BTZ) respectively did not result in elevated accumulation of CDC73 (Figs. 6C and S7A), indicating that CDC73, unlike β-catenin, is not directly degraded by UBR5 through UPS. Moreover, treatment with the autophagy inhibitor chloroquine (CQ) or bafilomycin A1 (BafA1) did not increase the level of CDC73 protein either (Fig. S7B, C), ruling out the possibility that CDC73 is degraded by the autophagy-lysosome pathway. Notably, reduction of CDC73 protein was observed only in the presence of UBR5 with either the broad-spectrum protein kinase inhibitor staurosporine (STS) (Fig. 6D) or the highly selective ERK2 inhibitor VX-11e (Ki < 2 nM) in a dose-dependent manner [28] (Fig. 6E). Interestingly, the degradation of CDC73 in 4T1/GFP cells treated with VX-11e was prevented with the additional treatment of MG132 (Fig. 6F). These results were then corroborated by *Erk2-*targeted siRNA (si*Erk2*) treatment (Fig. 6G). Moreover, the interaction of UBR5 with CDC73 was augmented by the VX-11e treatment in a co-IP assay (Fig. 6H). In a cell-based ubiquitination assay, Myc-tagged CDC73 was maximally polyubiquitinated by EGFP-tagged UBR5 in the presence of VX-11e (Fig. 6I). Hence, these results suggest that CDC73 is stabilized in a phosphorylated form via ERK2 and degraded in a non-phosphorylated form by UBR5’s E3 ubiquitin ligase activity via the 26S proteasome.

Given the importance of phosphorylation in CDC73’s stability, we then carried out a Phos-tag SDS-PAGE analysis to determine if the phosphorylation substrate of ERK2 is in CDC73 or UBR5. Treatment of 4T1 cells with VX-11e or si*Erk2* caused a clear reduction in phosphorylated CDC73 with little change in UBR5, compared to the untreated control (Fig. 6J, K), indicating that CDC73 is a phosphorylation substrate for ERK2.

ERK1 and 2 are both proline-directed kinases that preferentially catalyze the phosphorylation of substrates containing a Pro-Xaa-Ser/Thr-Pro sequence and the minimal consensus sequence is Ser/Thr-Pro [29, 30]. Thus, Thr^334^, Thr^359^, and Ser^465^ are possible phosphorylation sites in CDC73 for ERK1/2. We then constructed the three corresponding CDC73 mutants, T334A, T359A, and S465A, and tested their ability to be ubiquitinated by UBR5, which is a prerequisite step for target degradation, in a cell-based assay with or without VX-11e (Fig. 6L). Ubiquitination of CDC73 by UBR5 was markedly increased by the use of VX-11e. Among the three mutants, S465A retained the high ubiquitination level in the absence of VX-11e comparable to that of like CDC73 treated with VX-11e. By the Phos-tag SDS-PAGE assay, the level of phosphorylation of S465A was confirmed to have been strongly reduced compared to that CDC73 (Fig. 6M). Treatment of S465A with VX-11e had little effect on its heightened level of ubiquitination (Fig. 6N). Consistent with this property, S465A displayed a remarkably accelerated decaying rate compared to CDC73 in HEK293T cells treated with CHX (Fig. 6O). Taken together, these results demonstrate that Ser^465^ of CDC73 is the pivotal residue for phosphorylation by ERK2 and the protein stabilization against degradation by UBR5-mediated UPS.

### Lys^243^, Lys^247^ and Lys^257^ residues are critical for CDC73’s ubiquitination by UBR5

**Fig. 7:**
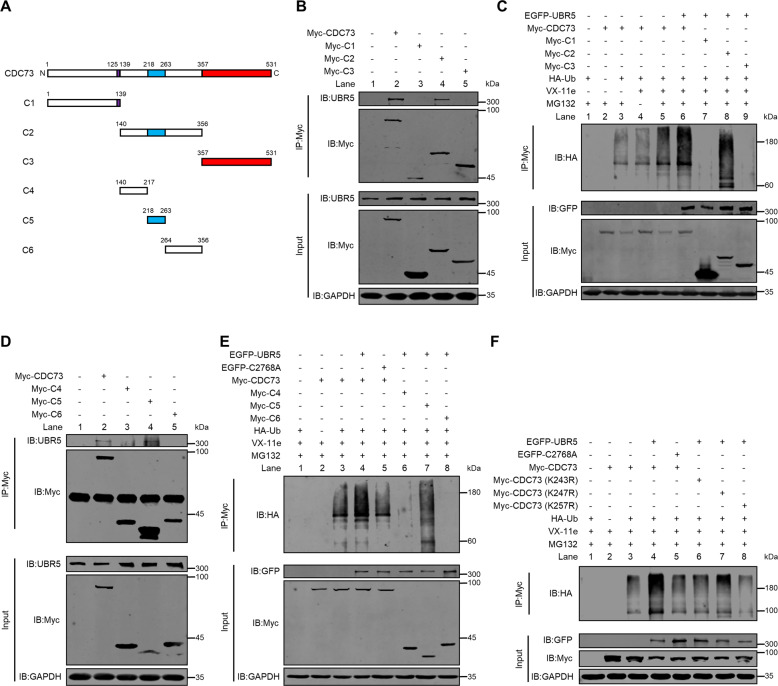
Identification of CDC73’s key residues for UBR5-mediated ubiquitination. **A** The schematic diagram of the full length and truncated fragments of CDC73. Co-IP with anti-Myc antibody analyzed the physical interaction of UBR5 with the full length or truncated fragments of CDC73 C1, C2, and C3 (**B**), and C4, C5, and C6 (**D**) in HEK293T cells transfected with the indicated plasmids in the presence of MG132 (10 μM, 8 h). Ubiquitination assays analyzed the ubiquitination level of the full length of CDC73 and its truncated fragments C1, C2, and C3 (**C**), and C4, C5, and C6 (**E**) modified by exogenous UBR5 under denaturing conditions in HEK293T cells transfected with the indicated plasmids and treated with vehicle, VX-11e (10 μM, 24 h) or/and MG132 (10 μM, 8 h). **F** Ubiquitination assays analyzed the ubiquitination level of WT and the mutants of CDC73 (K243A, K247A, and K257A) in HEK293T cells under the same condition as described in (**C**) and (**E**).

To identify the polyubiquitination domains of CDC73 (531 amino acids (aa)) modified by UBR5, we first constructed three truncated fragments with a C-terminus attached Myc-tag, C1 (aa 1-139), C2 (aa 140-356), and C3 (aa 357-531) (Fig. 7A) and tested their interaction and ubiquitination with UBR5. C2 was the only fragment out of the three that retained the UBR5-binding property like CDC73, as analyzed by co-IP (lane 4, Fig. 7B), and the ability to be ubiquitinated by UBR5 (lane 8, Fig. 7C). We then further truncated C2 into C4 (aa 140-217), C5 (aa 218-263), and C6 (aa 264-356) (Fig. 7A), and again tested their ability to interact with and be ubiquitinated by UBR5. The results showed that C5 was the key region for UBR5-binding (lane 4, Fig. 7D) and UBR5-mediated ubiquitination (lane 7, Fig. 7E). Since C5 contains three lysine (K) residues that can serve as potential sites for ubiquitination, we further constructed three Lys to Arg mutants, K243R, K247R, K257R, and assessed their ability to be ubiquitinated by UBR5 (Fig. 7F). The result revealed that all three mutants could reduce the ubiquitination of CDC73 (compare lanes 6-8 to lane 4), although K257R exhibited the maximum reduction among them. These data indicate that Lys^243^, Lys^247^ and Lys^257^ are all involved in CDC73’s ubiquitination and degradation by UBR5-mediated UPS.

## Discussion

The profound oncogenic effects of UBR5 on many malignant tumors had been previously reported [5–10], including our previous study in TNBC and ovarian cancer [5–7]. However, the detailed molecular mechanisms and key regulatory molecules in TNBC were still unclear.

In this study, we present for the first time that CDC73 is the critical molecule for countering UBR5’s pivotal activities not only on TNBC tumor growth, but also on metastasis, the current major clinical problem in human cancer. In the 4T1 murine TNBC model, we observed that the increased CDC73 protein level in *Ubr5*^−/−^ cells leads to significant reduction on both tumor growth and metastasis, and this phenotype can be almost fully reversed to the WT when *Cdc73* was further knocked down, via the mechanism of regulating CD8^+^ T cells infiltration and apoptosis in TME and inhibiting the expression of β-catenin and E-cadherin, respectively. Hence, CDC73 functions like a tumor suppressor in TNBC as most previous studies showed [27, 31–34], instead of an oncogene as some reports suggested [24, 35]. Notably, clinical metadata analyses in this study validate that low expression of *CDC73* is associated with poor relapse free survival of breast cancer patients, as well as decreased levels of tumor-infiltrating CD8^+^ T cells. The expression of *CDC73* is weakly but significantly negatively correlated with immune checkpoint molecules of *LGALS9*, *VSIR*, and *CD276* which are involved in suppressing T cell associated responses [36–38]. It also has a weak but significant positive correlation with the expression of costimulatory molecules *TNFSF4*, *TNFSF18*, and *CD80* that are involved in T cell activation [39–41]. Thus, CDC73 may influence T cell-mediated immune responses by regulating the expression of such immune checkpoint molecules and costimulatory molecules. In addition, low expression of CDC73 has also been found associated with adverse pathological parameters in human breast cancer cases [42, 43]. Moreover, although the mRNA level of CDC73 in patients is statistically lower in TNBC than non-TNBC, there is an overlapping between them, and the regulation of UBR5 to CDC73 was also observed in non-TNBC cell line MCF-7, indicating that the function of CDC73 and UBR5 may be more wide spread in breast cancers, not limited to TNBC. Collectively, these findings uncover a central role of CDC73 in antagonizing UBR5’s tumorigenic activities in a cell-intrinsic manner for tumor metastasis and extrinsically for tumor growth involving the adaptive immune system.

Defining the enzymatic substrates of UBR5 is crucial for developing targeted cancer therapies [22]. In this study, by co-IP, co-localization, and ubiquitination analysis, we demonstrated that CDC73 is a bona fide substrate of UBR5’s E3 ligase. Together with PAF1, CTR9, LEO1, RTF1, and SKI8/WDR61, CDC73 composes the highly conserved PAF1C [23], which regulates transcriptional events and histone modifications that take place during cell growth and survival, and embryonic development [25, 26]. Interestingly, our results also showed that UBR5 can not only down-regulate the expression of the CDC73 in breast cancer cells, but also additional PAF1C subunits PAF1, CTR9, and LEO1. In this context, CDC73 appears to play a dominant role in determining the UBR5-regulated level of PAF1, CTR9, and LEO1, in agreement with a previous report in HeLa cells [27]. PAF1C regulates transcription pause release and extension in cell growth and survival [24, 26], thus, reduced PAF1C factors due to UBR5’s activity could cause alterations in gene expression that can result in dysregulation of developmental programs and loss of control of cell division leading to cancer in humans [44]. Hence, CDC73/PAF1C might be a potential target for developing targeted breast cancer therapies. However, future in-depth studies are necessary to further delineate the transcriptional role of UBR5 with PAF1C.

**Fig. 8:**
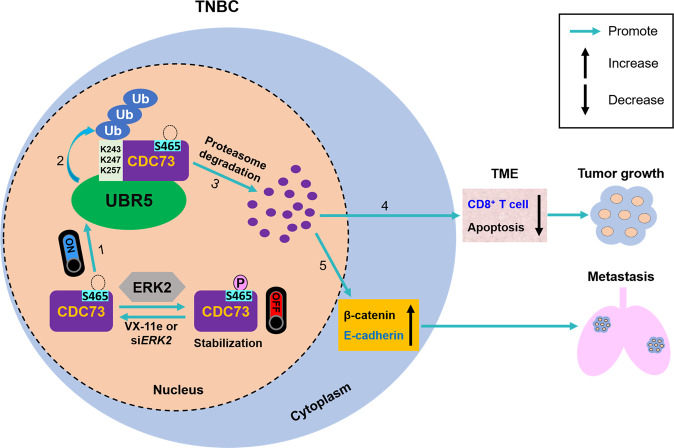
The molecular switch role and mechanism of CDC73 for UBR5-drived TNBC tumor growth and metastasis. In TNBC cells, CDC73 is stabilized by ERK2-mediated phosphorylation at Ser^465^. UBR5 targets non-phosphorylated CDC73 (1) and ubiquitinates it at Lys^243^, Lys^247^ and Lys^257^ residues (2) for degradation by UPS (3). Degradation of CDC73 promotes UBR5’s tumorigenic and immunoregulatory activities in TME (4), as well as tumor metastasis in a cell-intrinsic manner by regulating both mRNA and protein expression of β-catenin and E-cadherin (5).

The UPS is a subject of broad interests for its therapeutic potential in many cancers [45]. The stability of tumor suppressors is important for their function [46–48]. As a highly conserved HECT E3 ubiquitin ligase, UBR5 plays a key regulatory role in the UPS in cancer development [22]. Here, besides revealing CDC73 as a key substrate of UBR5, we further uncovered its ubiquitination and degradation by a seemingly non-canonical UPS mediated by UBR5. We observed, surprisingly, that CDC73’s protein stability is dependent on its phosphorylation by ERK2. Although the majority of target proteins are recognized and degraded by the UPS when phosphorylated, including p53 [49], PTEN [50], β-catenin [51], CBX4 [52], and CDH1 [53], a few examples have been reported in which the opposite is the case, such as Snail1 and ETV4, which are stabilized by ERK2-mediated phosphorylation with diminished ubiquitination [54, 55]. Hence, the phosphorylation state of CDC73 in TNBC could serve as a switch to trigger its degradation and further affect UBR5’s major cellular activities including apoptosis, epithelial-mesenchymal transition, and T cell infiltration. Given the protein kinases have the substrate specificity [56], here, we revealed that CDC73 is also a substrate of ERK2. Moreover, we have delineated the critical amino acid residues involved in CDC73’s turnover, i.e., Ser^465^ as the key residue for the phosphorylation of CDC73 via ERK2, and Lys^243^, Lys^247^, and Lys^257^ for CDC73’s ubiquitination by UBR5 (Fig. 8). Hence, one might seek to enhance tumor suppressor CDC73’s stability in cancer cells by maintaining its phosphorylated state, thus inhibiting its ubiquitination and degradation mediated by UBR5, as a potential therapeutic strategy for breast cancer patients with low CDC73 levels.

In summary, we provide the first evidence that CDC73 is a key substrate of UBR5 and demonstrate that CDC73 strongly antagonizes UBR5’s profound tumorigenic and metastatic activities via mechanisms of controlling CD8^+^ T cell-mediated anti-tumor response and increasing tumor cell apoptosis in TME, as well as via cell-intrinsic regulation of β-catenin and E-cadherin in tumor cells. Moreover, we reveal that CDC73 is stabilized when it is phosphorylated at Ser^465^ by ERK2 and destabilized by the non-canonical UPS mediated by UBR5 at the polyubiquitination sites of Lys^243^, Lys^247^ and Lys^257^ (Fig. 8). The molecular switch role of CDC73 in inhibiting UBR5’s pro-tumor capacities points to a novel potential therapeutic target and strategy for immune therapy of breast cancer.

## Materials and methods

### Cell lines and cell culture

4T1 and BT549 cell lines were purchased from the American Type Culture Collection (Manassas, VA, USA). MCF-7, MDA-MB-231, and HEK293T cell lines were purchased from the Cell Bank of the Chinese Academy of Sciences (Shanghai, China). 4T1 cell lines were cultured in RPMI 1640 medium (Gibco) supplemented with 10% FBS (Gibco) and 1% penicillin/streptomycin (Gibco). BT549, MCF-7, MDA-MB-231, and HEK293T cell lines were cultured in DMEM medium (Gibco) supplemented with 10% FBS and 1% penicillin/streptomycin. All cell lines were maintained at 37 °C in a humidified atmosphere containing 95% air and 5% CO_2_ with medium change every 2 days.

### Antibodies and reagents

Anti-UBR5 (1:200, sc-515494 and sc-9562), anti-CDC73 (1:200, sc-33638), anti-Actin (1:1000, sc-8432), and anti-GAPDH (1:1000, sc-47724) were purchased from Santa Cruz Biotechnology (Dallas, TX, USA). Anti-GFP (1:1000, M20004) and anti-HA (1:1000, M20003) were purchased from Abmart (Shanghai, China). Anti-ERK1/2 (1:1000, 4695), anti-cleaved caspase-3 (1:100, 9661), and anti-Myc (1:1000, 2276S) were purchased from Cell Signaling Technology (Danvers, MA, USA). Anti-cleaved PARP1 (1:100, ab32064) was purchased from Abcam (Cambridge, UK). Anti-E-cadherin (1:1000, 610181) and anti-β-catenin (1:1000, 610153) were purchased from BD Biosciences (San Jose, CA, USA). anti-PAF1 (1:1000, A300-172A) was purchased from Bethyl (Montgomery, TX, USA). Anti-CTR9 (1:1000, 21264-1-AP), anti-LEO1 (1:1000, 12281-1-AP), anti-WDR61 (1:1000, 22536-1-AP), and anti-RTF1 (1:1000, 12170-1-AP) were purchased from Proteintech (Wuhan, China). IRDye^®^ 800CW Donkey anti-Mouse IgG Secondary Antibody (1:10,000, 926-32210) and IRDye^®^ 800CW Donkey anti-Rabbit IgG Secondary Antibody (1:10,000, 926-32213) were purchased from LI-COR Biosciences (Lincoln, NE, USA). Anti-CD16/32 (1:200, 101302), PerCP/Cyanine 5.5 anti-mouse CD45 (1:500, 103131), APC anti-mouse CD3 (1:500, 100236), PE anti-mouse CD4 (1:500, 100408), FITC anti-mouse CD8a (1:500, 100705), PE anti-mouse CD49b (1:500, 108907), PerCP/Cyanine 5.5 anti-mouse Ly-6C (1:500, 128011), and FITC anti-mouse CD11b (1:500, 101206) were purchased from BioLegend (San Diego, CA, USA). APC anti-mouse Ly-6G (1:500, 2055150) was purchased from Invitrogen (Carlsbad, CA, USA). Cy3-labeled Donkey anti-Goat IgG (1:500, A0502), Alexa Fluor 488-labeled Goat anti-Mouse IgG (1:500, A0428), and Alexa Fluor 555-labeled Donkey anti-Rabbit IgG (1:500, A0453) were purchased from Beyotime (Shanghai, China).

Tunel assay kit (G1507) and H&E dye solution (G1003) were purchased from Servicebio (Wuhan, China). Phosbind acrylamide (F4002) was purchased from APExBIO (Houston, TX, USA). SIGMAFAST™ protease inhibitor tablets (S8820), PhosSTOP™ phosphatase inhibitor tablets (4906845001), puromycin (540411), polybrene (TR-1003-G), STS (19-123-M), CQ (C6628), Collagenase D (11088866001), DNase I (10104159001), and Dispase® II (D4693) were purchased from Sigma-Aldrich (St. Louis, MO, USA). VX-11e (HY-14178), MG132 (HY-13259), BTZ (HY-10227), and BafA1 (HY-100558) were purchased from MCE (Monmouth Junction, NJ, USA). Protein A/G PLUS-Agarose (sc-2003) was purchased from Santa Cruz Biotechnology (Dallas, TX, USA). Cell Lysis Buffer for western blotting or IP (P0013), DAPI (C1002), and Cell Counting Kit-8 (C0038) were purchased from Beyotime (Shanghai, China). 3-color Regular Range Protein Marker (GF6616) was purchased from GeneFirst (Oxfordshire, UK). Spectra™ Multicolor High Range Protein Ladder (26625), Alexa Fluor^TM^ 488 Annexin V/Dead Cell Apoptosis Kit (V13241), TRIzol (15596026), Lipofectamine 2000 reagent (11668019), RNAiMAX Transfection Reagent (13778100), and BCA kit (23227) were purchased from Thermo Fisher Scientific (Waltham, MA, USA). PrimeSTAR® GXL DNA Polymerase (R050Q) was purchased from Takara Biotechnology (Tokyo, Japan). The HiScript II Q RT SuperMix for qPCR (+gDNA wiper) kit (R223-01) was purchased from Vazyme (Nanjing, China). Hieff® qPCR SYBR Green Master Mix (No Rox) (11201ES03) was purchased from Yeasen Biotechnology (Shanghai, China).

### Plasmids, siRNA, and transient transfections

The pCDH-EGFP plasmid was constructed by inserting EGFP into the vector of pCDH-CMV-MCS-EF1-puro (System Biosciences). The plasmid expressing N-terminal EGFP-tagged human UBR5 (EGFP-UBR5) was constructed by cloning the human *UBR5* gene into pCDH-EGFP. The EGFP-UBR5 C2768A mutant plasmid was purchased from Addgene (#52051, Watertown, MA, USA). The plasmid expressing mCherry was constructed by inserting mCherry into the vector of pcDNA3.1 (+) (Invitrogen). The plasmids expressing C-terminal Myc-tagged human CDC73 (Myc-CDC73) and its truncated fragments (C1–C6) were constructed by cloning the full length or truncated fragments (C1–C6) of human *CDC73* gene into pcDNA3.1-mCherry, respectively. The pCMV-HA-Ub plasmid was purchased from Miaolingbio (#P0554, Wuhan, China). All the plasmids were confirmed by sequencing.

The siRNA targeting mouse *Erk2* (5′-GCUCUGCUUAUGAUAAUCUTT-3′) was purchased from GenePharma (Shanghai, China).

According to the manufacturer’s instructions, the expressing plasmids and siRNAs were transient transfected into cells by Lipofectamine 2000 reagent or RNAiMAX Transfection Reagent, respectively.

### Generation of single-site mutants of CDC73

Three single-site mutants of Myc-tagged CDC73 T334A, T359A, and S465A were designed for key phosphorylation residues analysis. And three single-site mutants of Myc-tagged CDC73 K243R, K247R, and K257R were designed for critical ubiquitination residues analysis. All the mutants were generated by one-step PCR-based site-directed mutagenesis with PrimeSTAR® GXL DNA Polymerase according to the manufacturer’s instructions. All mutants were then confirmed by sequencing. The primers used in the site-directed mutagenesis were shown in Table S1.

### shRNAs, lentiviral production, and infection

The shRNAs were constructed by inserting the corresponding sequences into pLKO.1-TRC Cloning Vector (#10878, Addgene) following the manufacturer’s protocol. The sequence of mouse *Cdc73*-targeted shRNA (sh*Cdc73*) is 5′-GGGTCACGGACACCCATTATT-3′. The sequence of human *UBR5*-targeted shRNA 1 (sh*UBR5* #1) is 5′-GCTGTAGATTTCAACTTAGAT-3′, and shRNA 2 (sh*UBR5* #2) is 5′-TTGGAACAGGCTACTATTAAA-3′. The sequence of human *CDC73*-targeted shRNA 1 (sh*CDC73* #1) is 5′-CCTAGAATGATGTGTTTCTAT-3′, and shRNA 2 (sh*CDC73* #2) is 5′-GCGTCAACATCGGCAAGTATA-3′. All shRNAs were validated by sequencing.

The lentiviruses were generated by co-transfection of HEK293T cells with psPAX2, pMD2.G, and the corresponding shRNA using Lipofectamine 2000 reagent following the manufacturer’s instructions. The supernatants containing the lentivirus were collected and filtered through a 0.45 μm syringe filter at 24 and 48 h post transfection. Cells were then infected with lentivirus for 24 h in the presence of polybrene (8 μg/ml) and selected with puromycin.

### Mice, tumor growth and metastasis models

Female BALB/c mice (8-week-old) were purchased from Beijing Vital River Laboratory Animal Technology Co., Ltd (Beijing, China). All mouse studies have been approved by the Institutional Animal Care and Use Committee of Shanghai Jiao Tong University.

For the 4T1 tumor growth model, a total of 5 × 10^5^ cells were subcutaneously injected into the inguinal mammary fat pad of BALB/c mice. Tumor size was monitored every 2 days with an electronic caliper and tumor volume was calculated as volume = length × width^2^ × 0.5. Mice were sacrificed on day 28 after injection and the tumors and spleens were harvested for further analysis [6].

For the 4T1 lung metastasis model, a total of 2 × 10^5^ cells were intravenously injected into BALB/c mice through tail vein. Mice were sacrificed on day 21 after injection and the lungs were collected for further analysis [6].

### Flow cytometry

The tumors harvested from the tumor-bearing mice were digested with tissue dissociation buffer [Collagenase D (1 mg/ml), DNase I (50 μg/ml), and Dispase® II (1 U/ml) in PBS] by periodic vortexing for 1 h in a 37 °C water bath. The spleens were mashed and the erythrocytes were removed by ACK lysis buffer. Cell suspensions from the tumors and spleens were then strained with 70 μm filters. The single-cell suspensions were incubated in mouse Fc block (anti­CD16/32) and then stained with the appropriate surface antibodies in FACS Buffer as described previously [6].

### 6-thioguanine assay

Lungs in the mice tumor metastasis model were excised, minced, and digested in tissue dissociation buffer with periodic vortexing for 1 h in a 37 °C water bath and then the erythrocytes were removed by ACK lysis buffer. Cell suspensions were strained with 70 μm filters. The single-cell suspensions were then seeded in 6-well plates and cultured for 7 to 14 days in the presence of 6-thioguanine (60 mmol/l), followed by fixing with 4% paraformaldehyde and staining with 0.5% crystal violet. The number of colonies formed in each well was counted and photographed under the microscope.

### TUNEL, H&E, and IHC staining

All specimens were fixed with 4% formalin in phosphate buffer for 24 h and then embedded in paraffin, and sectioned at 4 μm. The sections were then deparaffinized at 60 °C for 30 min, cleared in 100% xylene, and rehydrated with a graduated series of ethanol solutions from 100 to 70%.

For TUNEL and H&E staining, tumor sections were subjected to TUNEL staining using Tunel assay kit and the lung sections were stained with H&E dye solution following the manufacturer’s protocol.

For IHC staining, tumor sections were treated with citrate buffer (pH 6.0) for antigen retrieval. After inhibition of endogenous peroxidase activity for 25 min with 3% H_2_O_2_, the sections were incubated with 3% BSA buffer for 30 min for blocking of non-specific binding. The sections were then incubated with rabbit anti-cleaved caspase-3 (1:100) or anti-cleaved PARP1 (1:100) at 4 °C overnight and then treated with HRP conjugated secondary antibody for 1 h at room temperature. The sections were further stained with DAB chromogenic reagent and counterstained with hematoxylin.

At least five random fields of views per section were captured by the upright Fluorescent Microscope (Axio Imager M2) and the percentage of apoptotic cells, cleaved caspase-3-positive cells, and cleaved PARP1-positive cells was analyzed by ImageJ software.

### Cell apoptosis assay

Cell apoptosis was analyzed by using the Alexa Fluor^TM^ 488 Annexin V/Dead Cell Apoptosis Kit (Thermo Fisher Scientific) according to the manufacturer’s instructions by flow cytometry. In brief, cells were collected, washed in cold phosphate-buffered saline (PBS), and suspended in 1X annexin-binding buffer. Then, cells were stained with 5 μl Alexa Fluor® 488 annexin V and 1 μl propidium iodide at room temperature for 15 min in the dark, and analyzed by flow cytometry.

### Transwell migration and invasion assay

For the migration assay, 5 × 10^4^ cells in serum-free medium were seeded into the upper chambers and 500 μl of medium with 10% FBS was added to the lower chambers. After incubation for 24 h, the cells that had migrated to the lower surface of the transwell membrane were fixed with 4% paraformaldehyde and stained with 0.5% crystal violet.

For the invasion assay, 5 × 10^4^ cells in serum-free medium were seeded into the upper chambers containing Matrigel bedding (Corning) and 500 μl of medium supplemented with 10% FBS was added to the lower chambers. After incubation for 24 h, the cells that had invaded to the lower surface of the transwell membrane were fixed with 4% paraformaldehyde and stained with 0.5% crystal violet.

Several random fields of views of above assays were captured by the Upright-Reverse Fluorescent Microscopy (Revolve & ECHO) and counted for each membrane.

### RNA isolation and quantitative real-time PCR (qPCR)

Total RNA was extracted with TRIzol reagent, and cDNA was prepared with the HiScript II Q RT SuperMix for qPCR (+gDNA wiper) kit (Vazyme). cDNA was mixed with Hieff® qPCR SYBR Green Master Mix (Yeasen Biotechnology) and subjected to qPCR with a sequence detection system (CFX; Bio-Rad Laboratories). The primers used in the qPCR were shown in Table S2.

### Western blotting

Cells were collected and lysed with cell lysis buffer (Beyotime) supplemented with protease inhibitor (Sigma-Aldrich) for 30 min on ice. The lysates were centrifuged at 12,000 × *g* for 10 min at 4 °C, and supernatants were collected. Protein concentration was quantified with a BCA kit (Thermo Fisher Scientific). A total of 50 μg of proteins were separated by 10% SDS-polyacrylamide gel and transferred to a nitrocellulose membrane. After blocking with 5% milk, the membranes were incubated first with the primary antibodies, and then with fluorescent secondary antibodies. The membrane was finally detected by the LI-COR Odyssey CLx imaging system (LI-COR Biosciences) and quantified by normalization to GAPDH with ImageJ software.

### Co-IP

A total of 1000 μg of cellular extracts from 4T1 or HEK293T cells were incubated with the primary antibody or IgG plus 40 µl of resuspended Protein A/G PLUS-Agarose on a rocker platform at 4 °C overnight. Pellets were then washed four times with lysis buffer and the precipitated proteins were eluted from the pellets by resuspending the pellets in 2 × SDS-PAGE loading buffer and boiling for 10 min. The boiled immune complexes were then subjected to western blotting analysis.

### Immunofluorescence

4T1 cells grown on coverslips were fixed with 4% paraformaldehyde at room temperature for 30 min, permeabilized with 0.5% Triton X-100 at room temperature for 30 min, blocked with 5% BSA at room temperature for 1 h, and then incubated with the primary antibodies overnight at 4 °C, and followed by staining with the secondary antibodies at room temperature for 2 h in the dark. DAPI (Beyotime) was used to stain the nuclei of cells for 5 min. Images were documented by the upright Fluorescent Microscope (Axio Imager M2).

### Phosphorylation analysis

Cells were lysed with cell lysis buffer (Beyotime) supplemented with protease inhibitor (Sigma-Aldrich) and phosphatase inhibitor (Sigma-Aldrich) for 30 min on ice. A total of 30 μg of proteins were subjected to Phos-tag SDS-PAGE according to the manufacturer’s instructions. In total, 6% SDS-polyacrylamide Phos-tag (50 μM) gels and 3% SDS-polyacrylamide (strengthened with 0.5% agarose) Phos-tag (50 μM) gels were used to separate phosphorylated and non-phosphorylated CDC73 and UBR5, respectively [57].

### Ubiquitination analysis under denaturing conditions

Cells were collected and lysed with 100 μl cell lysis buffer (2% SDS, 150 mM NaCl, 10 mM Tris-HCl, pH 8.0) supplemented with 2 mM sodium orthovanadate, 50 mM sodium fluoride, and protease inhibitor (Sigma-Aldrich). The cellular extracts were boiled for 10 min, sheared with a sonication device, and incubated with 900 μl of dilution buffer (10 mM Tris-HCl, pH 8.0, 150 mM NaCl, 2 mM EDTA, 1% Triton) at 4 °C for 60 min with rotation, followed by high-speed centrifugation (20,000 × *g*) for 30 min at 4 °C, and supernatants were then collected [58]. A total of 1000 μg of proteins were subjected to co-IP with the anti-Myc antibody, followed by western blotting analysis with anti-HA antibody.

### Statistical analysis

Data analysis was performed using GraphPad Prism 5.0 software, and presented with error bars as mean ± SEM. One way ANOVA and two-tailed unpaired Student’s *t* test were used to compare experimental groups. Statistical significance was defined as a *p* value of <0.05. Levels of significance were indicated as *p < 0.05; **p < 0.01; ***p < 0.001; ns, not significant.

## Supplementary information

Supplemental material

Original Data File

Checklist

## Data Availability

The data of *CDC73* mRNA expression in human breast cancer were obtained from Gene Expression Omnibus (GEO) at GSE42568 and GSE76275. The correlation of *CDC73* expression level with relapse free survival of breast cancer patients (*n* = 2032) and lymph node-positive breast cancer patients (*n* = 814) were evaluated by using the Kaplan–Meier plotter database (http://www.KMplot.com). The correlation of *CDC73* expression level with the infiltration level of CD8^+^ T cells in the tumor microenvironments (TME) of human breast cancer was analyzed by using the Immune Estimation Resource (TIMER2.0) database (*n* = 1100) (http://timer.cistrome.org/). Correlation analysis of mRNA expression between *CDC73* and *LGALS9*, *VSIR*, *CD276*, *TNFSF4*, *TNFSF18*, and *CD80* in human breast cancer from TCGA (*n* = 1084) was performed by using the cBioPortal database (https://www.cbioportal.org/). All other data needed to evaluate the conclusions in the paper are present in the paper and/or the Supplementary Materials. Additional data related to this paper may be requested from the authors.
