# Supplementary material for: UBR5 targets tumor suppressor CDC73 proteolytically to promote aggressive breast cancer
Source: Cell Death Dis. 2022 May 12;13(5):451. doi: 10.1038/s41419-022-04914-6 (PMC9098409; doi:10.1038/s41419-022-04914-6)
Supplement: Supplementary file 1 — Supplemental material [file 41419_2022_4914_MOESM1_ESM.docx]

Supplementary Information for

**UBR5 targets tumor suppressor CDC73 proteolytically to promote aggressive breast cancer**

Gang Xiang^1^, Shuxuan Wang^1^, Ling Chen^1^, Mei Song^2^, Xiaoxu Song^1^, Huan Wang^1^, Pengbo Zhou^3^, Xiaojing Ma^2^, Jing Yu^1^

This file contains the following contents:

Supplementary Figures and Figure Legends

Supplementary Tables

**
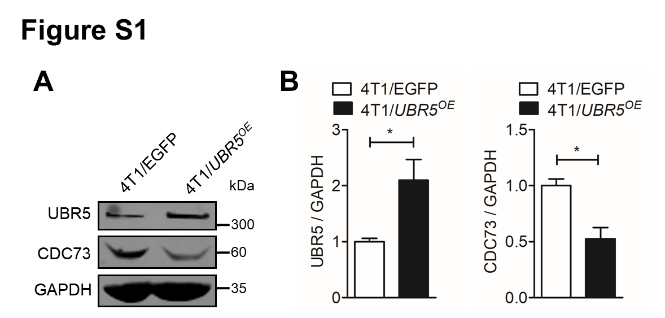
Supplementary Figures and Figure Legends**

**Supplementary Figure S1. Ectopic expression of UBR5 in 4T1 cells inhibits the protein level of CDC73.** **A** The protein levels of UBR5 and CDC73 were detected by western blotting in control (4T1/EGFP) and 4T1 exogenously expressing human *UBR5* (4T1/*UBR5*^OE^) cells. **B** The quantification of the protein level of UBR5 and CDC73 in **A** was shown as mean ± SEM for triplicates; **P* < 0.05.


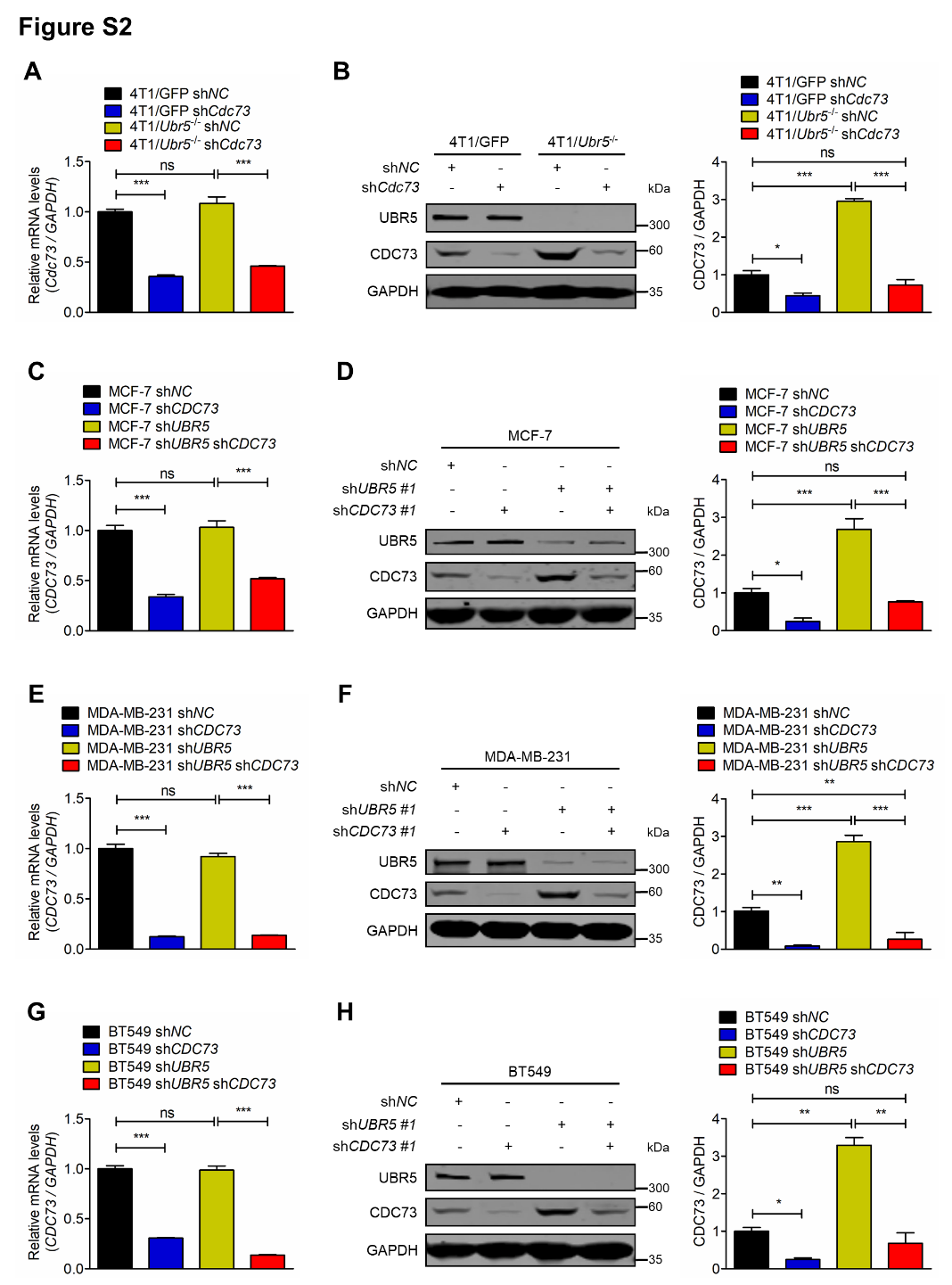
**Supplementary Figure S2. The knockdown efficiency of *CDC73* in 4T1, MCF-7, MDA-MB-231, and BT549 breast cancer cells via *CDC73*-targeted shRNAs*.* A, C, E, G** mRNA level was monitored by qPCR of each cell line**. B, D, F, H** Protein level was monitored by western blotting of each cell line. Data were shown as mean ± SEM for triplicates; ns, not significant; *P < 0.05; **P < 0.01; ***P < 0.001.

**
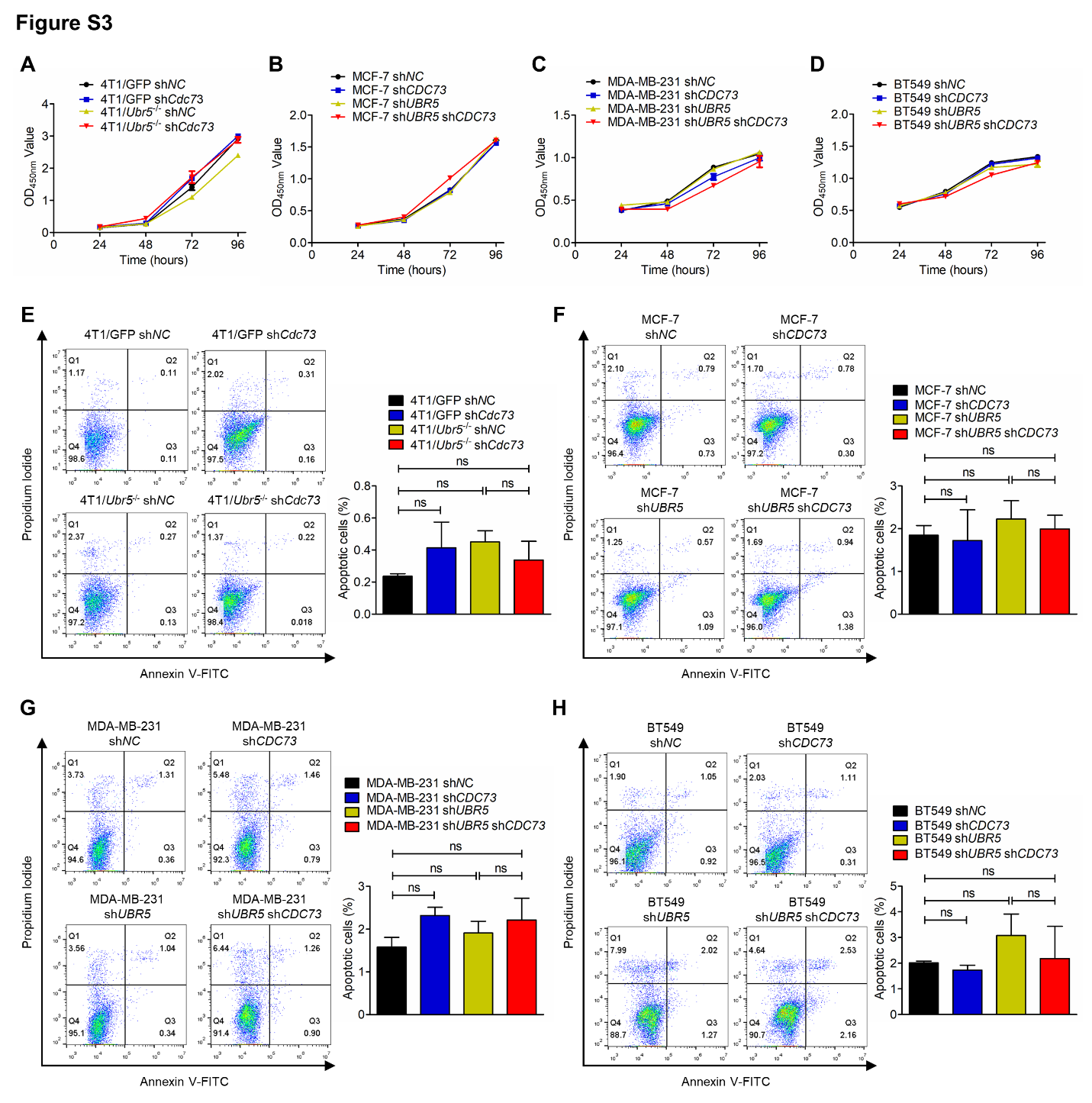
Supplementary Figure S3. Effects of CDC73 on cell proliferation and apoptosis in 4T1, MCF-7, MDA-MB-231, and BT549 breast cancer cells. A-D** Cell growth of each cell line was measured by cell counting kit-8 (CCK-8) assay *in vitro*. **E-H** Cell apoptosis of each cell line was analyzed by flow cytometry. Data were shown as mean ± SEM for triplicates; ns, not significant.


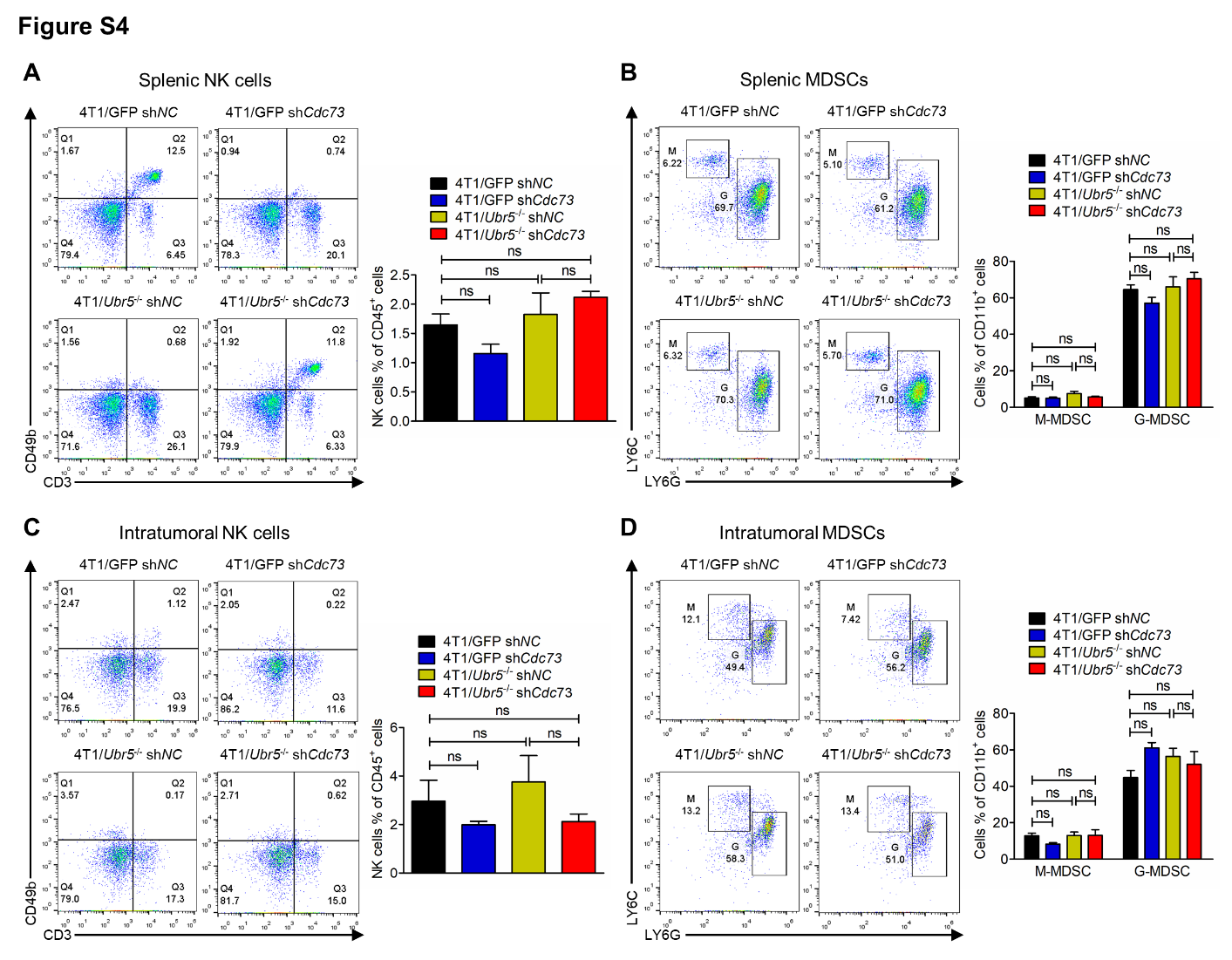
**Supplementary Figure S4. Representative FACS images and quantification of splenic NK cells (A), splenic MDSCs (B), intratumoral NK cells (C), and intratumoral MDSCs (D) in murine TNBC growth model.** Female BALB/c mice were injected with 5 × 10^5^ 4T1/GFP sh*NC*, 4T1/GFP sh*Cdc73*, 4T1/*Ubr5*^-/-^ sh*NC*, and 4T1/*Ubr5*^-/-^ sh*Cdc73* cells, respectively, into the mammary fat pad and sacrificed on day 28 after injection (n = 5). Data were shown as mean ± SEM for triplicates; ns, not significant.

**
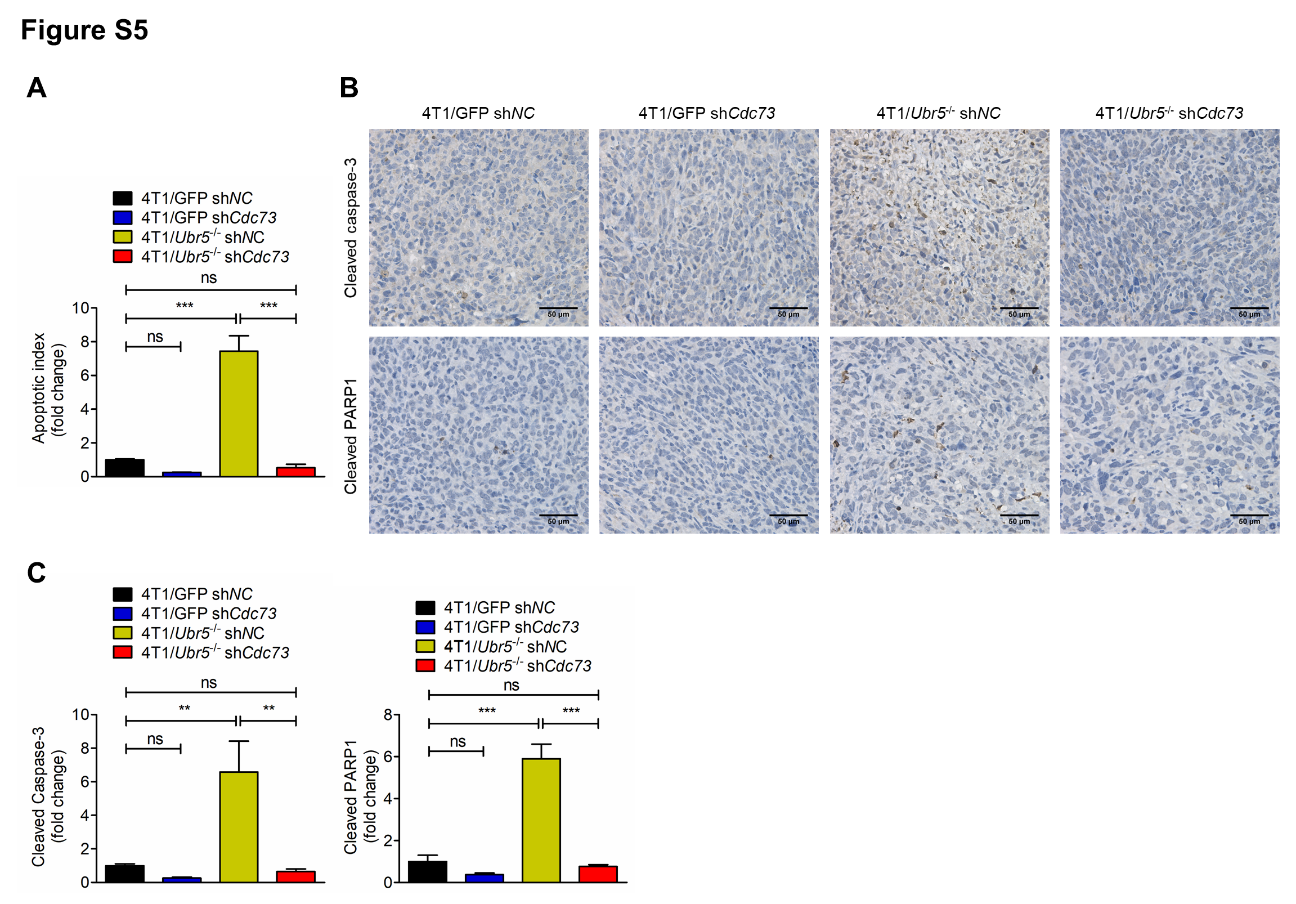
Supplementary Figure S5. The effects of CDC73 and UBR5 on cell apoptosis *in vivo*. A** The quantification of TUNEL staining of the tumor sections in **Fig. 3H** (n = 3). **B, C** The expression of cleaved caspase-3 and cleaved PARP1 in tumors was examined by IHC staining and quantified by ImageJ software (n = 3). Data were shown as mean ± SEM; ns, not significant; ***P* < 0.01; ****P* < 0.001.


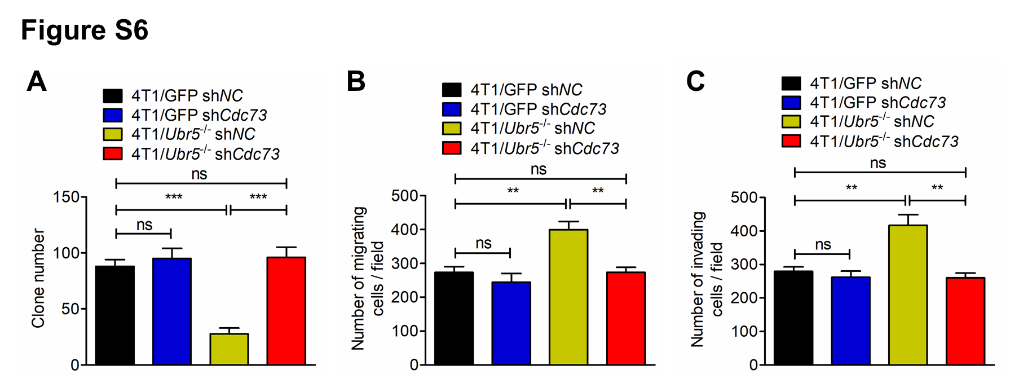
**Supplementary Figure S6.** **Quantitation of the effects of CDC73 of 4T1/GFP sh*NC*, 4T1/GFP sh*Cdc73*, 4T1/*Ubr5*^-/-^ sh*NC*, and 4T1/*Ubr5*^-/-^ sh*Cdc73* cells on cell clonogenicity (A), migratory (B) and invasive capacities (C) in Figure 4E-G**. Data were presented as mean ± SEM; ns, not significant; ***P* < 0.01; ****P* < 0.001.


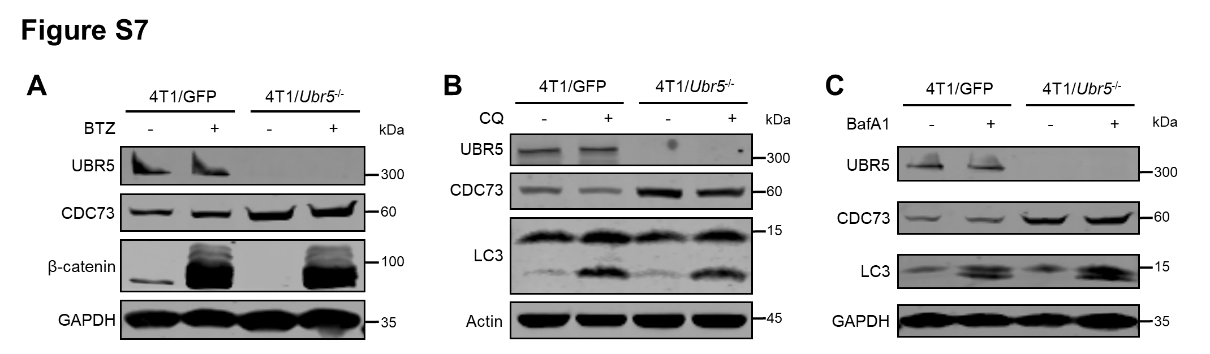
**Supplementary Figure S7. CDC73 was not directly degraded by UBR5 through UPS or the autophagy-lysosome pathway. A-C** The protein level of CDC73 was detected by western blotting in both 4T1/GFP and 4T1/*Ubr5^-/-^* cells treated with vehicle or BTZ (50 nM, 8 h) (**A**), CQ (25 μM, 24 h) (**B**), and BafA1 (25 nM, 12 h) (**C**).

**Supplementary Tables**

**Supplementary Table S1. Site-directed mutagenesis primers used in the study.**

| T334A | 5’-TGCATCTGCCCGGAAGACTCAGGCTCCTGCAG-3’ (forward)  5’-TACTGGCTGGGCTGCAGGAGCCTGAGTCTTCC-3’ (reverse) |
| --- | --- |
| T359A | 5’-AATCAGAAGAAAGGATCTCGAGCACCCATTATCA-3’ (forward)  5’-GCAGGAATTATGATAATGGGTGCTCGAGATCCT-3’ (reverse) |
| S465A | 5’-CCATGGCTTTTGCCTGATGGAGCACCAGTTGA-3’ (forward)  5’-TAGCAAATATATCAACTGGTGCTCCATCAGGCAAA-3’ (reverse) |
| K243R | 5’-AACTATCTTACAAAGCACAGGAAGGAATTTTTCCA-3’ (forward)  5’-CAAAAATGTTCTTGGAAAAATTCCTTCCTGTGCTT-3’ (reverse) |
| K247R | 5’-AGCACAGGAAAGAATTTTTCCAAGAACATTTTTGC-3’ (forward)  5’-AGATTGAAGAATTGCAAAAATGTTCCTGGAAAAATT-3’ (reverse) |
| K257R | 5’-ACATTTTTGCAATTCTTCAATCTGTAAGAGCCAGAGAA-3’ (forward)  5’-ACGCCCTTCTTCTCTGGCTCTTACAGATTGAAG-3’ (reverse) |

**Supplementary Table S2. qPCR primers for gene expression used in the study.**

| m*Cdc73* | 5’-GGGGAAGCATCAACATCAGCA-3’ (forward)  5’-GCTCTTTTGACTTGAGTAGACCG-3’ (reverse) |
| --- | --- |
| m*Ubr5* | 5’-GTCCATCCATTTCGTGGTCCA-3’ (forward)  5’-GGGTGGCTGTTCAAATTGTACTT-3’ (reverse) |
| m*β-catenin* | 5’-CCTAGCTGGTGGACTGCAGAA-3’ (forward)  5’-CACCACTGGCCAGAATGATGA-3’ (reverse) |
| m*E-cadherin* | 5’-CTGTCAATAGGGACACCGGG-3’(forward)  5’-TGTGCTCAAGCCTTCACCTT-3’ (reverse) |
| m*GAPDH* | 5’-AGGTCGGTGTGAACGGATTTG-3’ (forward)  5’-TGTAGACCATGTAGTTGAGGTCA-3’ (reverse) |
| h*CDC73* | 5’-TCCGACGTGCAGCTACTGA-3’ (forward)  5’-CGATGTTGACGCTTCACCATTG-3’ (reverse) |
| h*UBR5* | 5’-CCAGACAGATTGGAATTGGGTAA-3’ (forward)  5’-CATGGAGAGTCGCTTGTCCT-3’ (reverse) |
| h*GAPDH* | 5’-GGAGCGAGATCCCTCCAAAAT-3’ (forward)  5’-GGCTGTTGTCATACTTCTCATGG-3’ (reverse) |
