# Supplementary material for: UBR5 targets tumor suppressor CDC73 proteolytically to promote aggressive breast cancer
Source: Cell Death Dis. 2022 May 12;13(5):451. doi: 10.1038/s41419-022-04914-6 (PMC9098409; doi:10.1038/s41419-022-04914-6)
Supplement: Supplementary file 2 — Original Data File [file 41419_2022_4914_MOESM2_ESM.pdf]

Original Data for

# UBR5 targets tumor suppressor CDC73 proteolytically to promote aggressive triple-negative breast cancer

Gang Xiang, Shuxuan Wang, Ling Chen, Mei Song, Xiaoxu Song, Huan Wang, Pengbo Zhou, Xiaojing Ma, Jing Yu

This file contains the following contents:

Original WB images

Original immunofluorescence images

Original cell/tissue morphology, tumor and lung tissue images

# Original WB images

Figure 2A

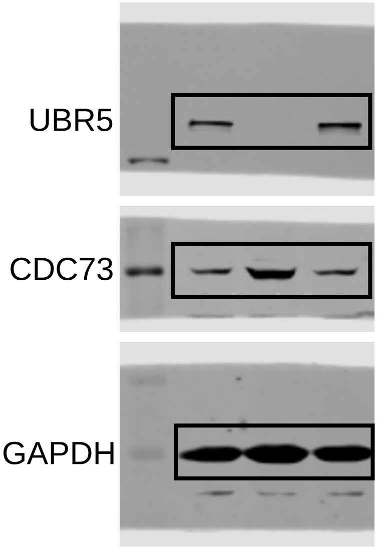


Figure 2B

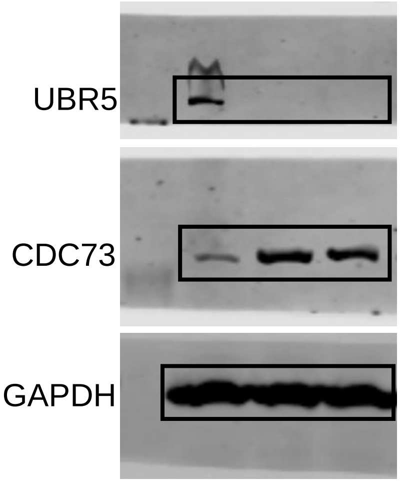


Figure 2C

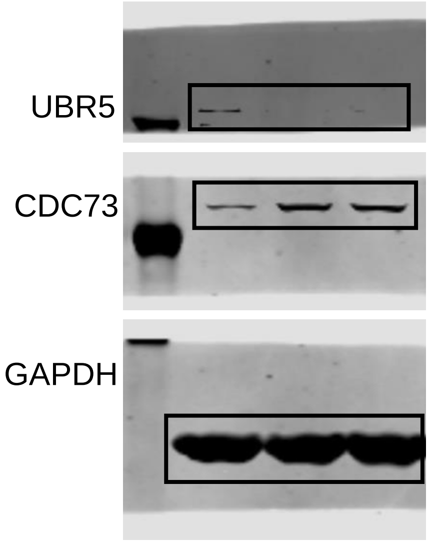


Figure 2D

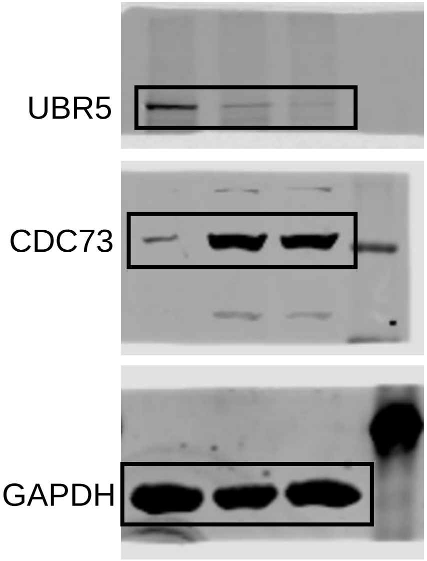


Figure 4B

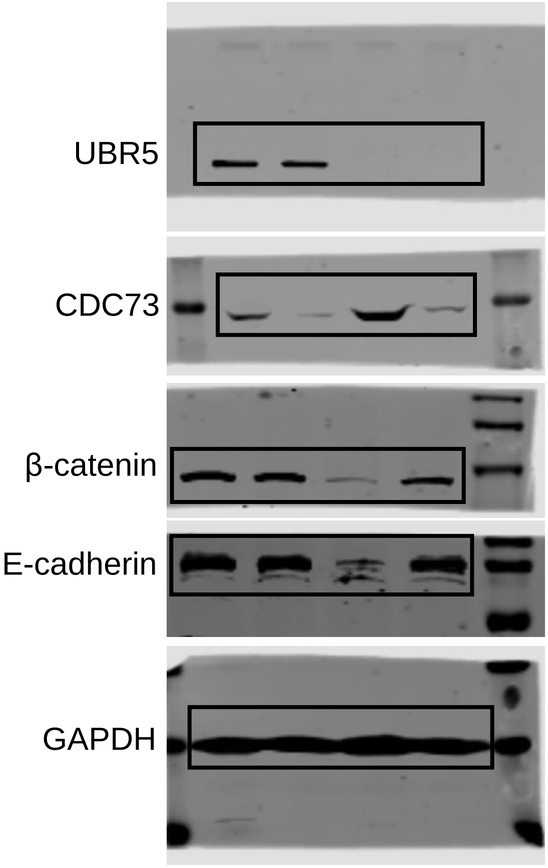

Figure 5A

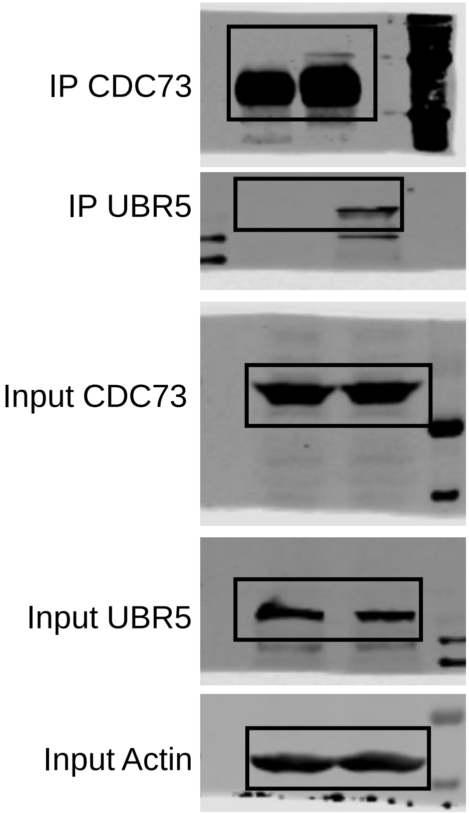


Figure 5D

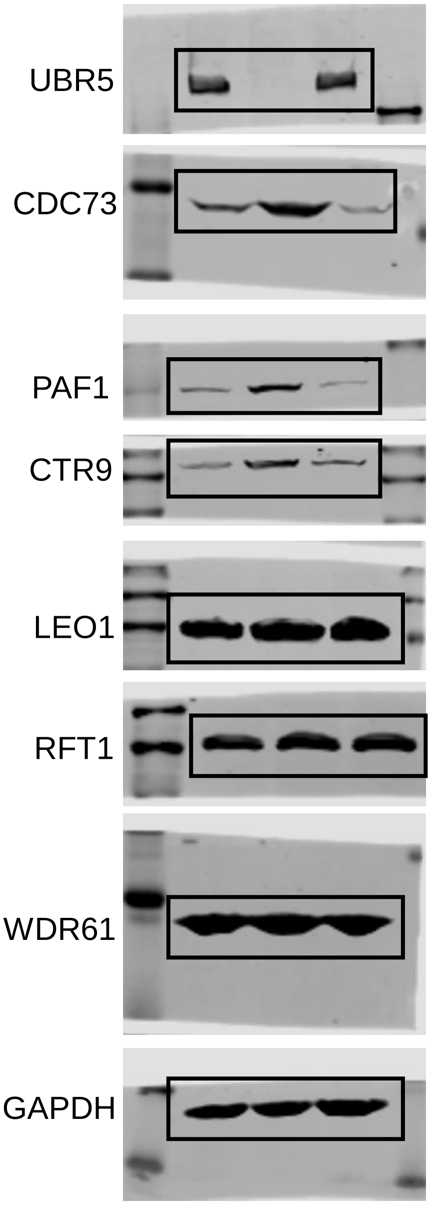


Figure 5E

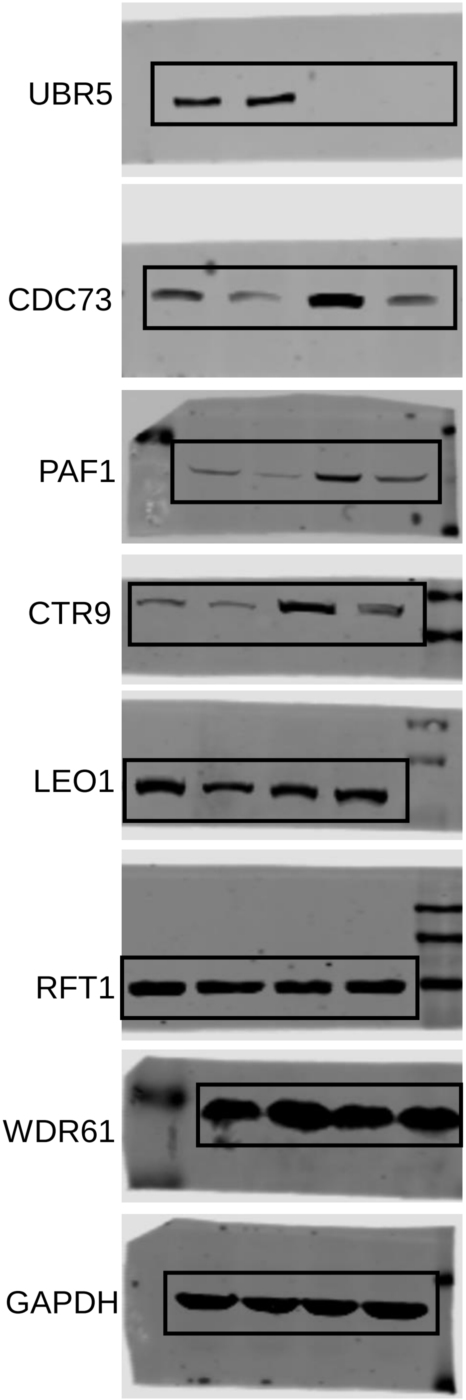


Figure 5B

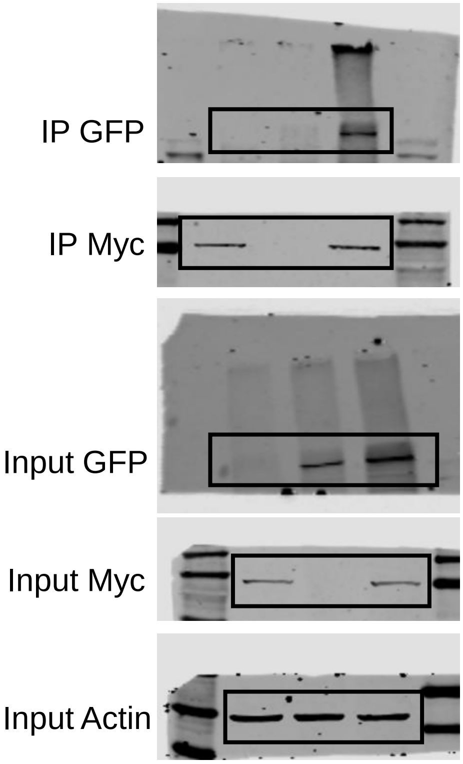

Figure 5F

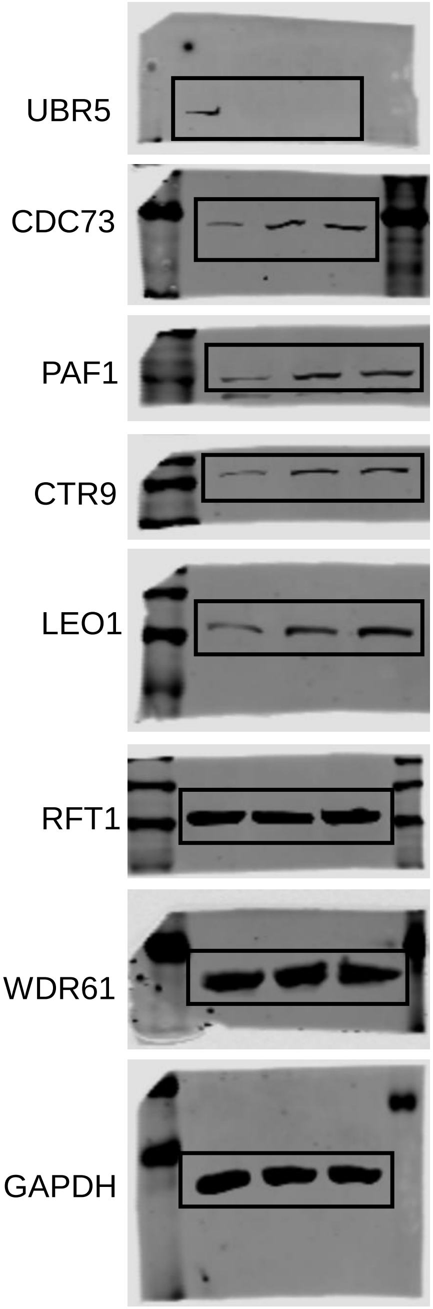


Figure 5G

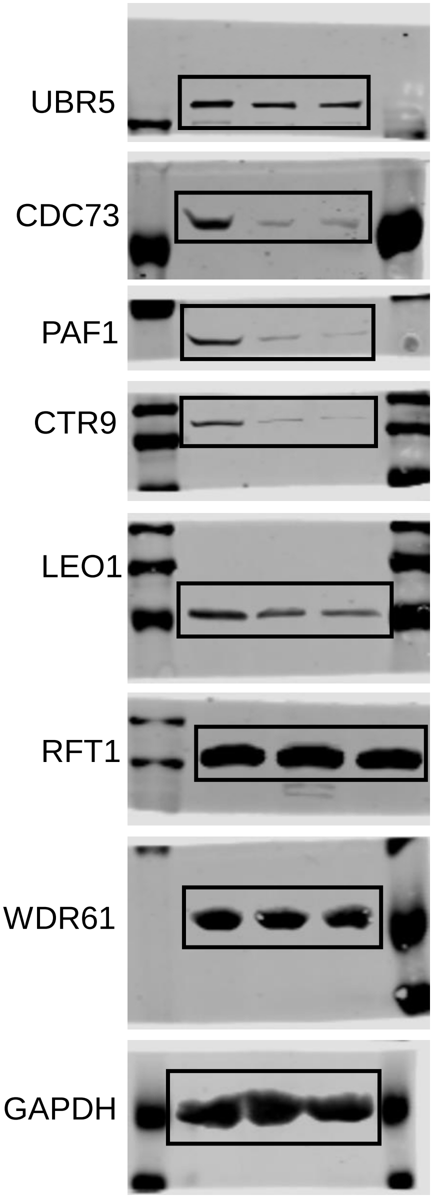


Figure 6A

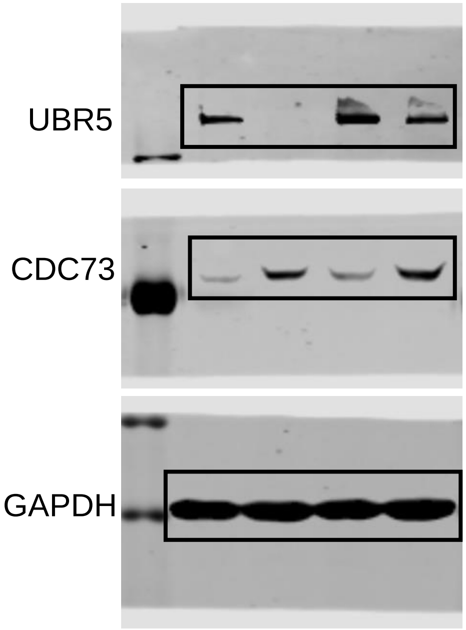

Figure 6B

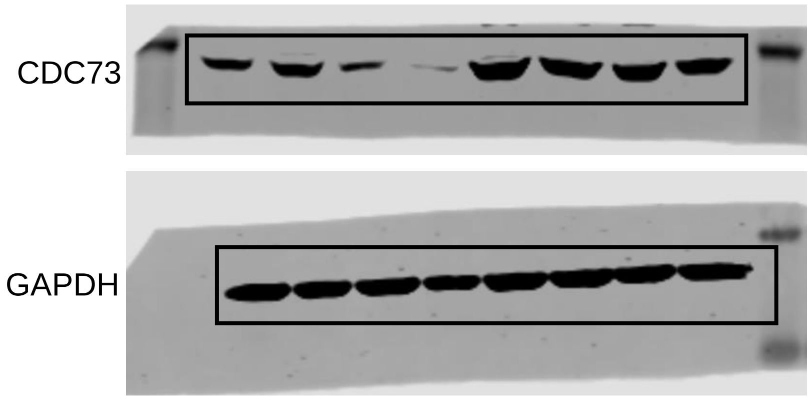


Figure 6D

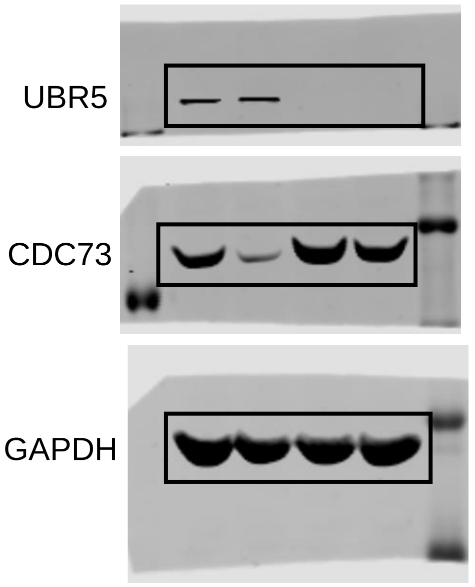


Figure 6C

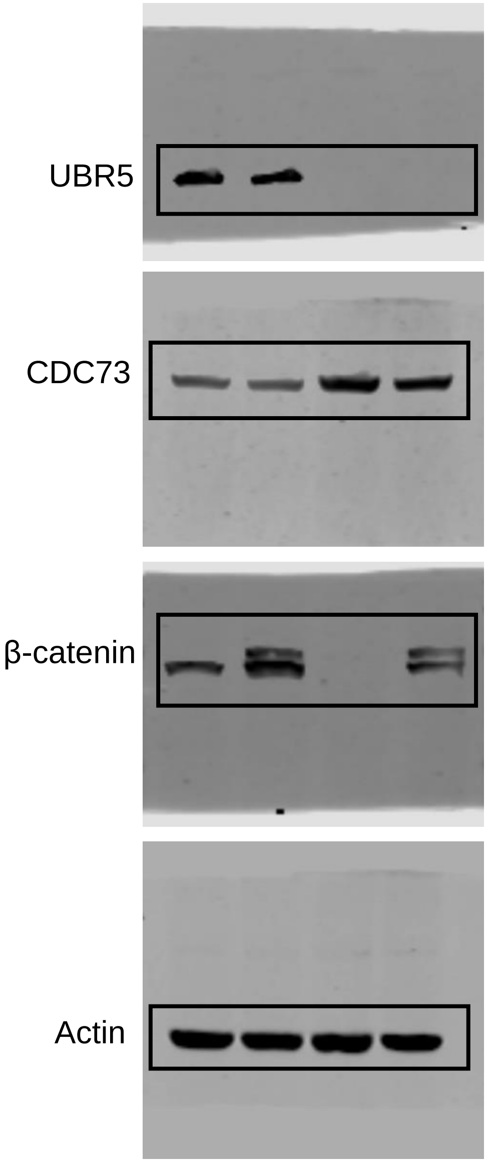


Figure 6E

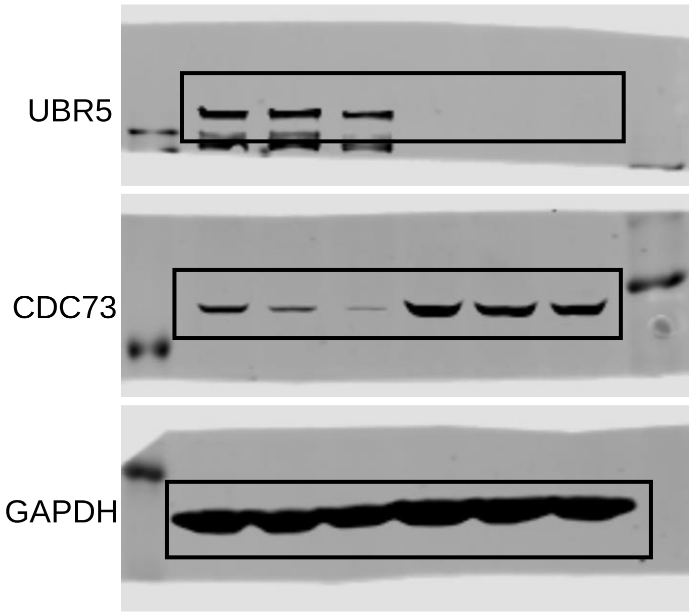

## Figure 6F

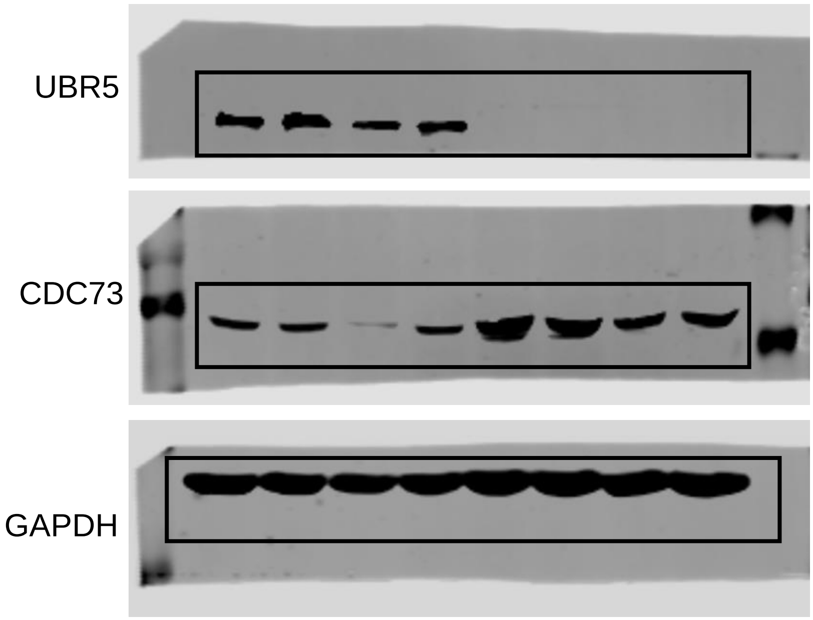


## Figure 6G

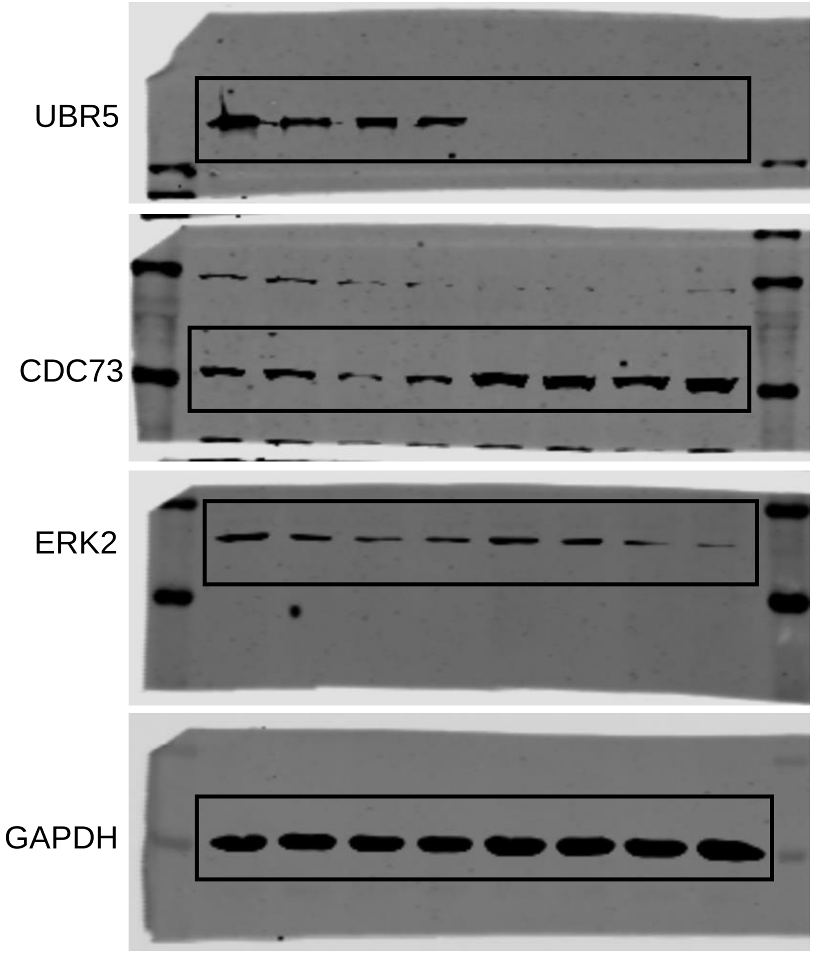


## Figure 6H

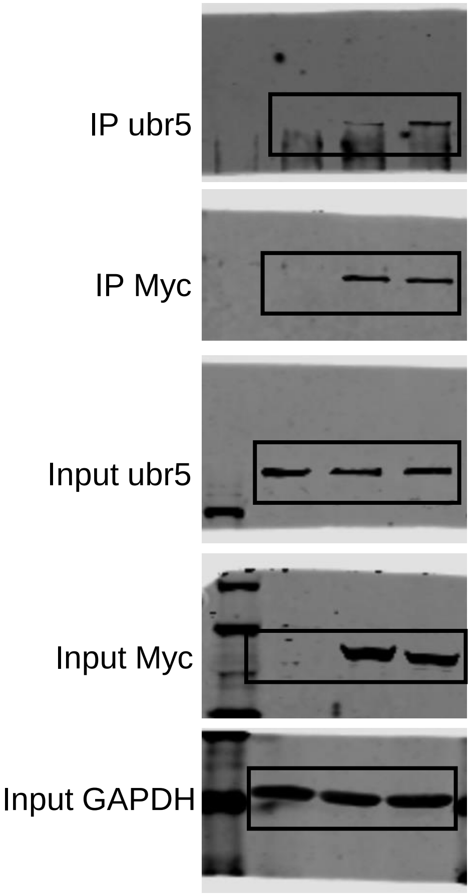

Figure 6I

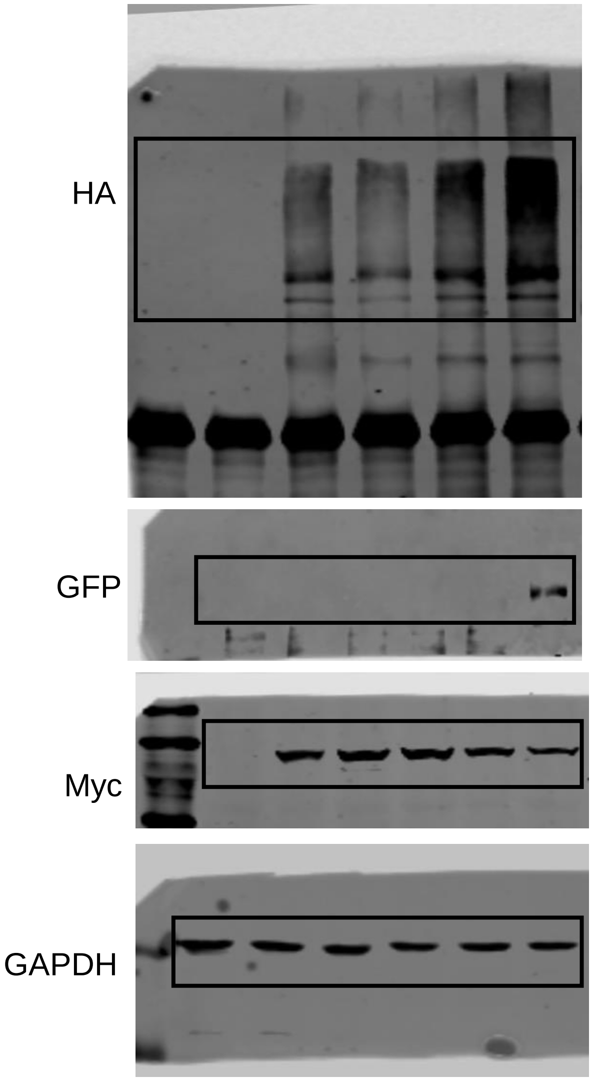


Figure 6J

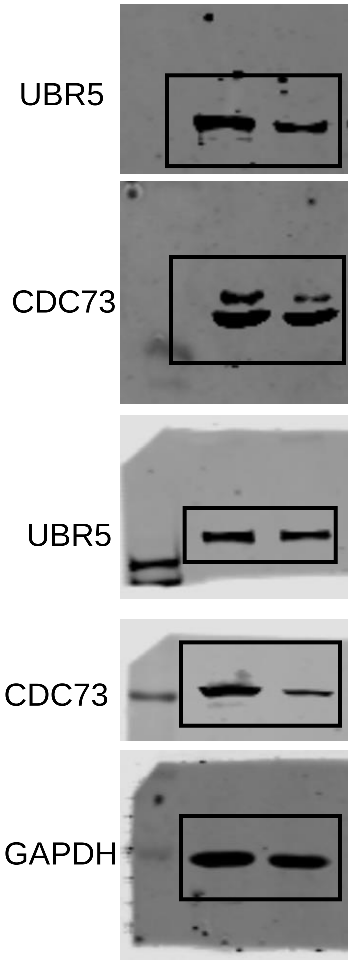


Figure 6K

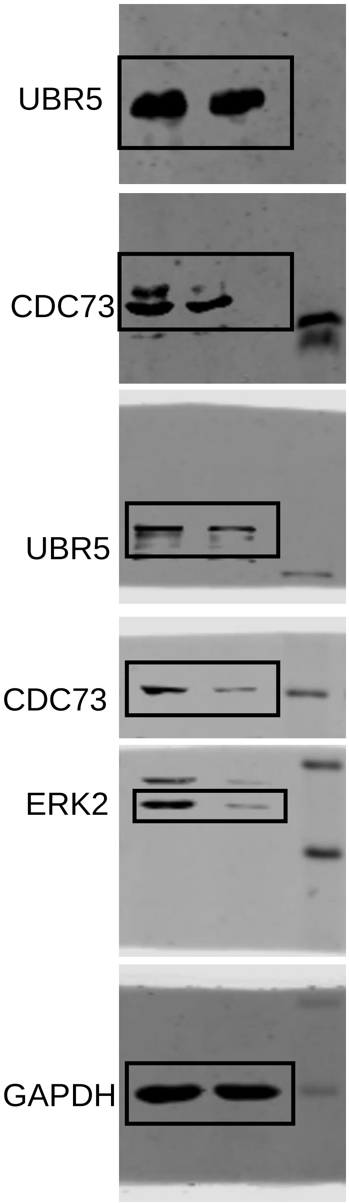

## Figure 6L

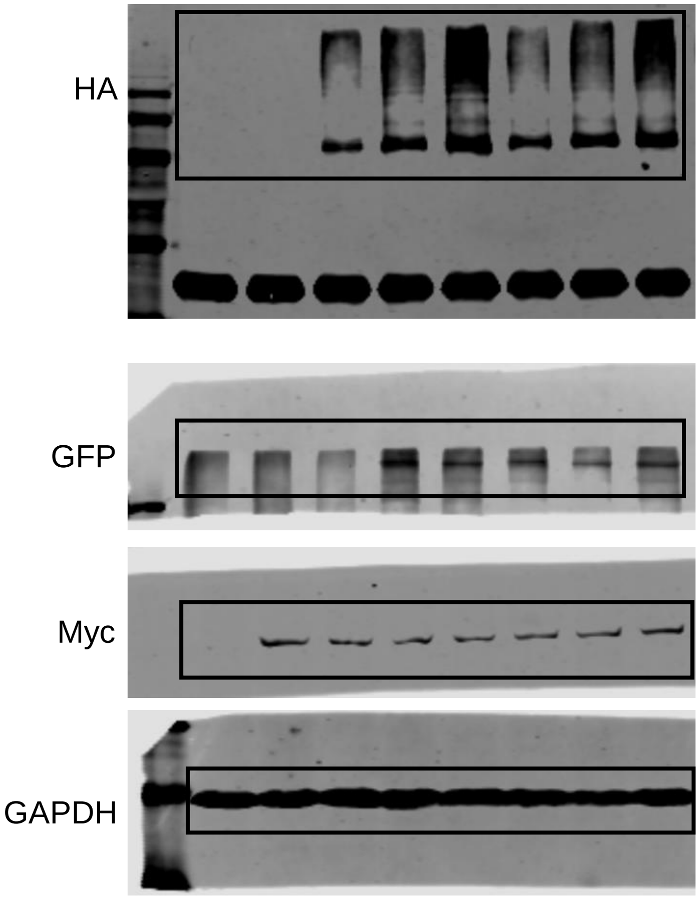


## Figure 6M

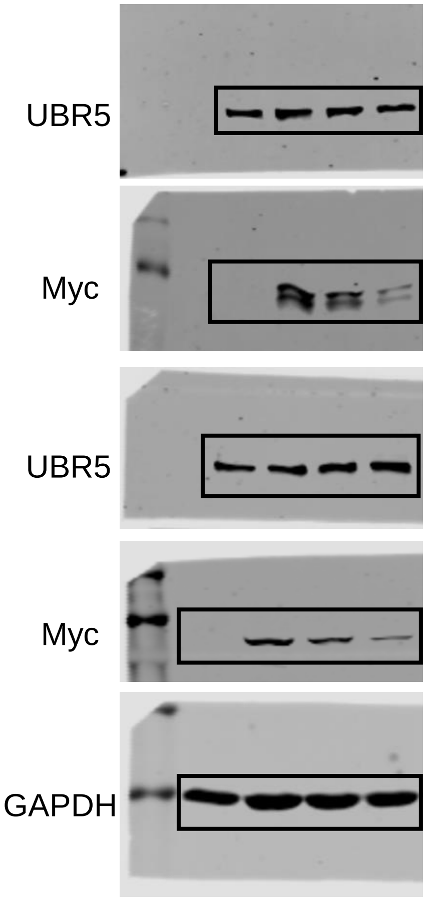


## Figure 6N

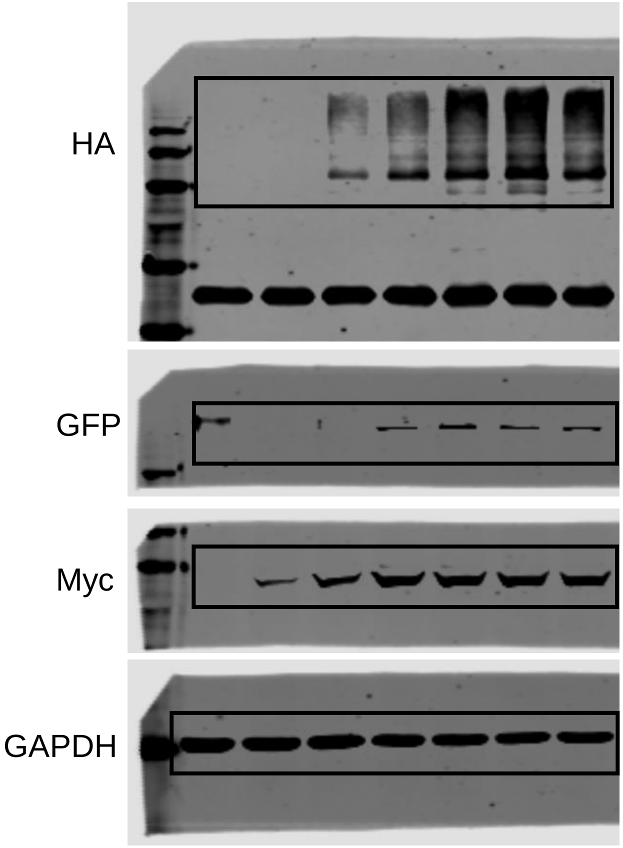


## Figure 6O

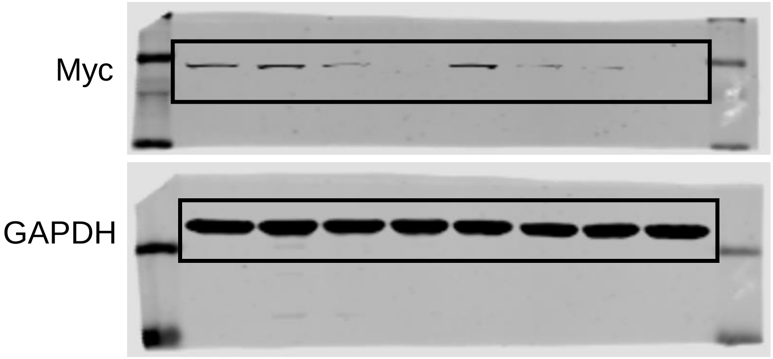

# Figure 7B

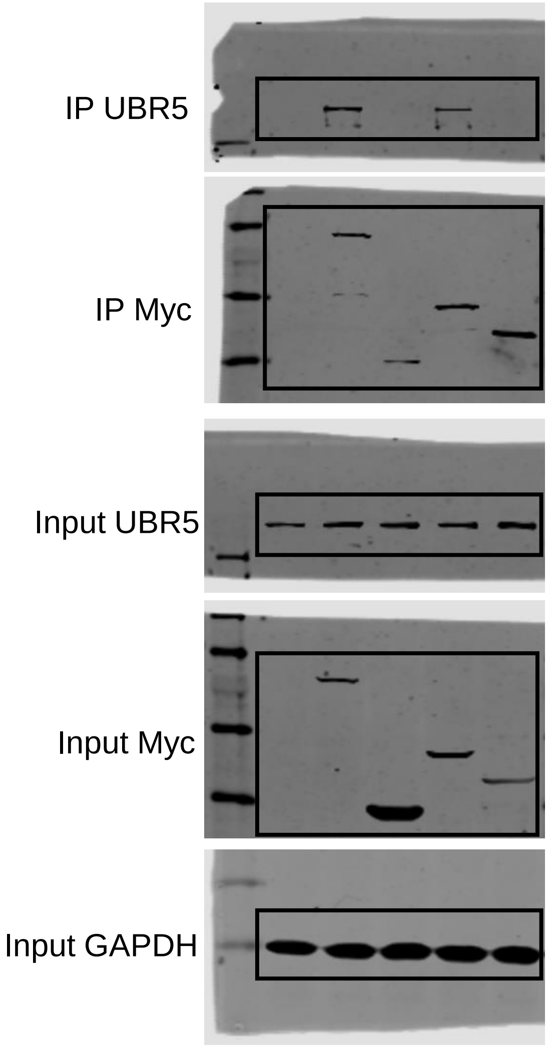


# Figure 7C

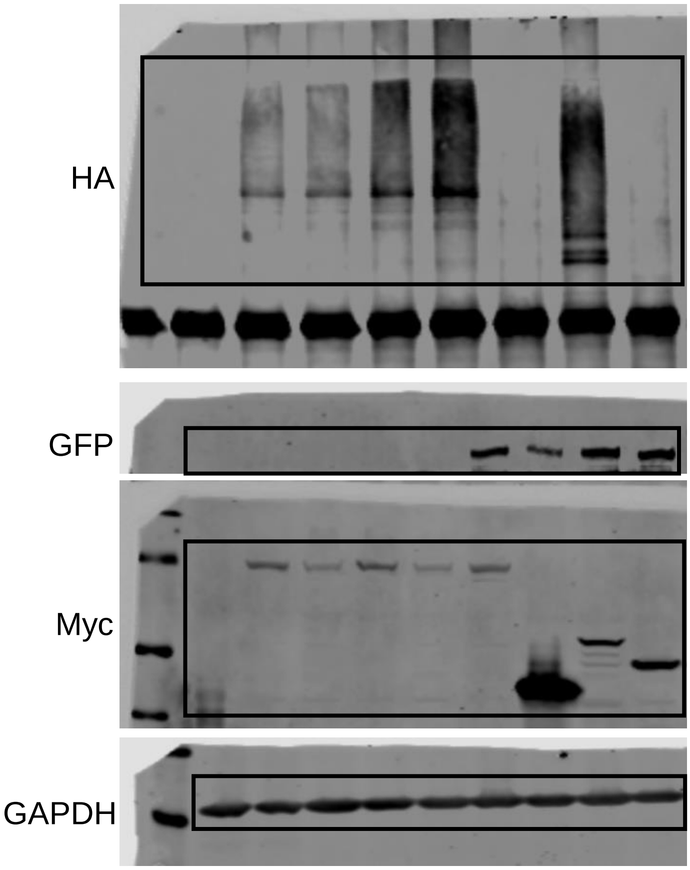

## Figure 7D

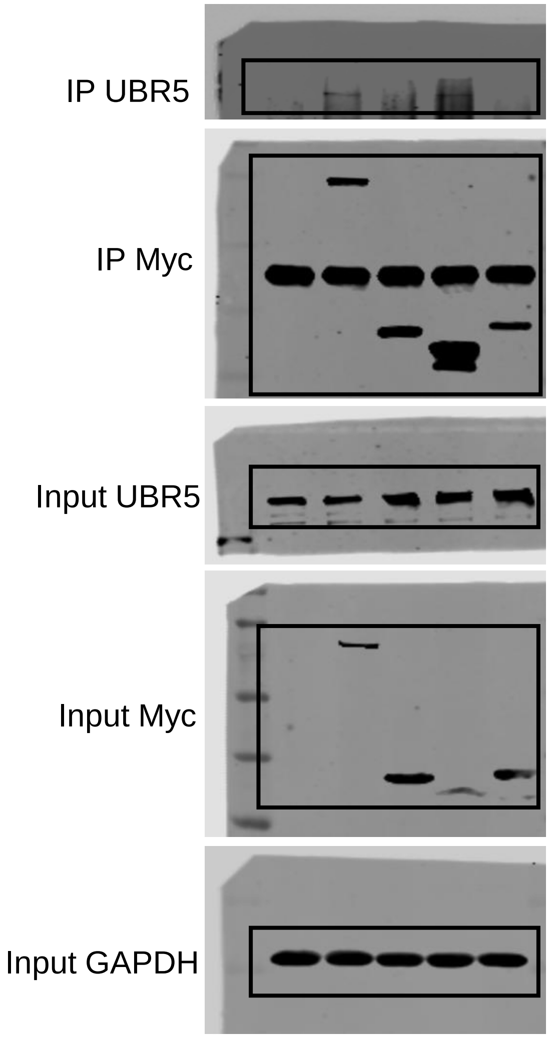


## Figure 7E

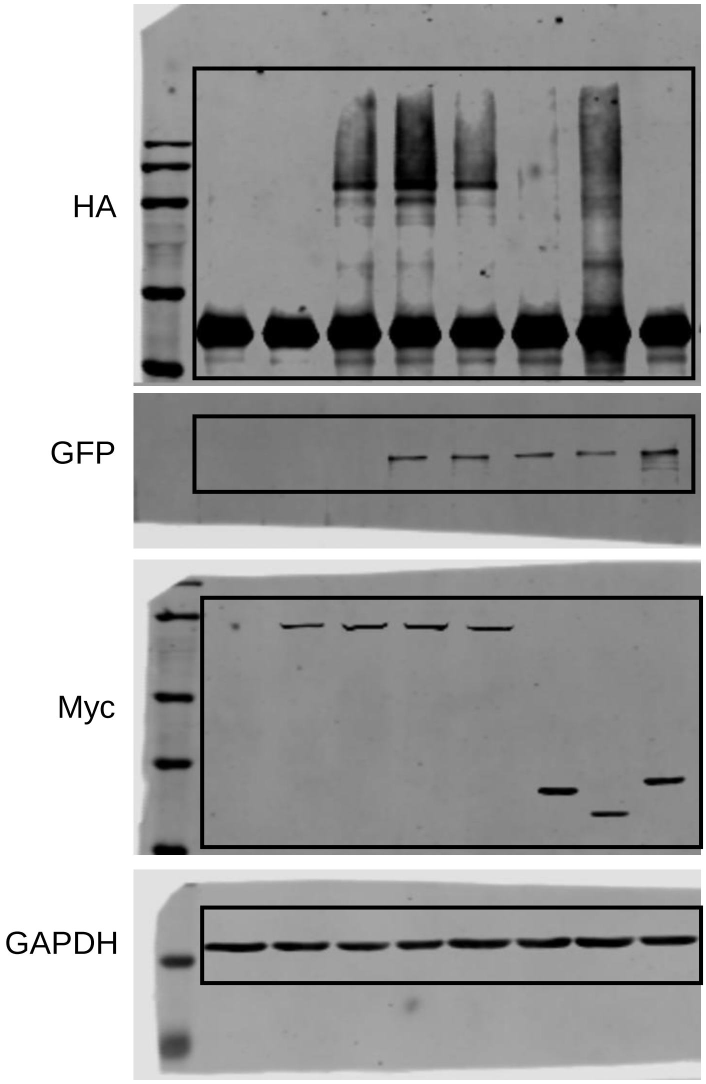


## Figure 7F

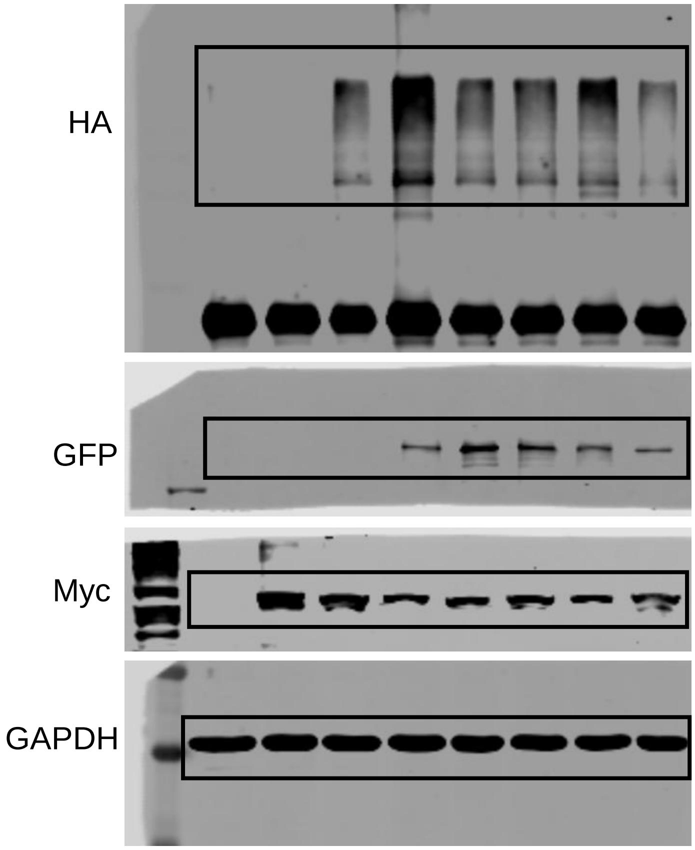

## Figure S1A

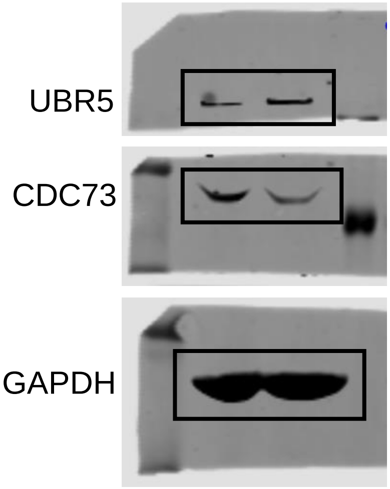


## Figure S2B

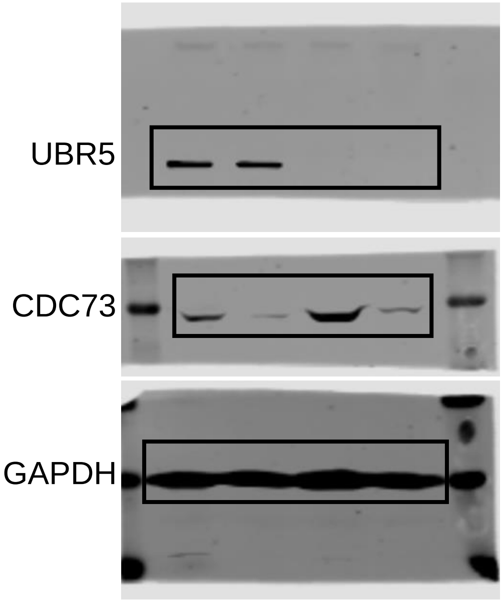


## Figure S2D

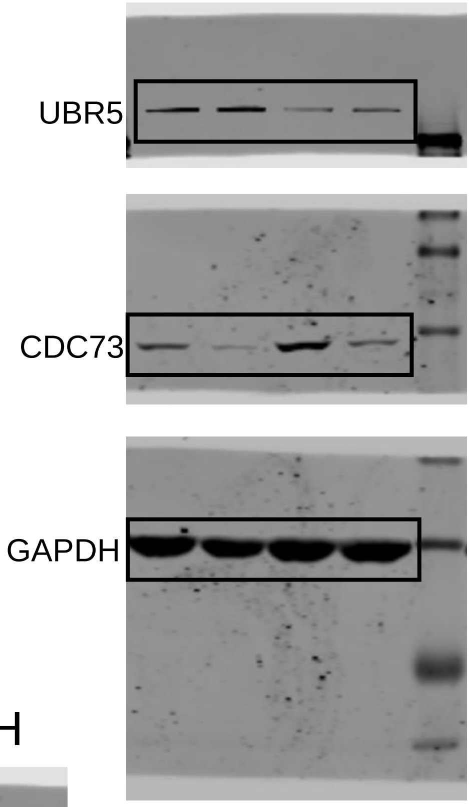


## Figure S2F

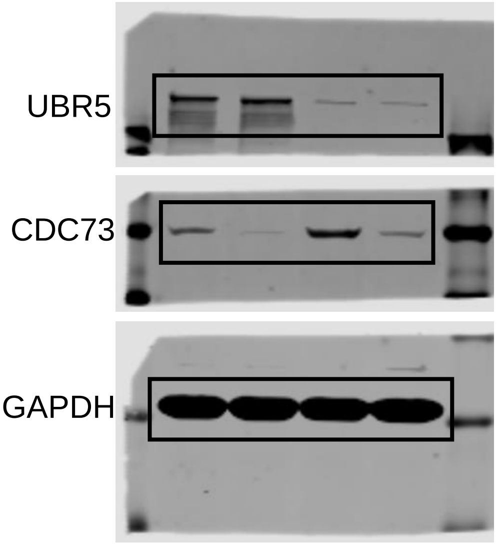


## Figure S2H

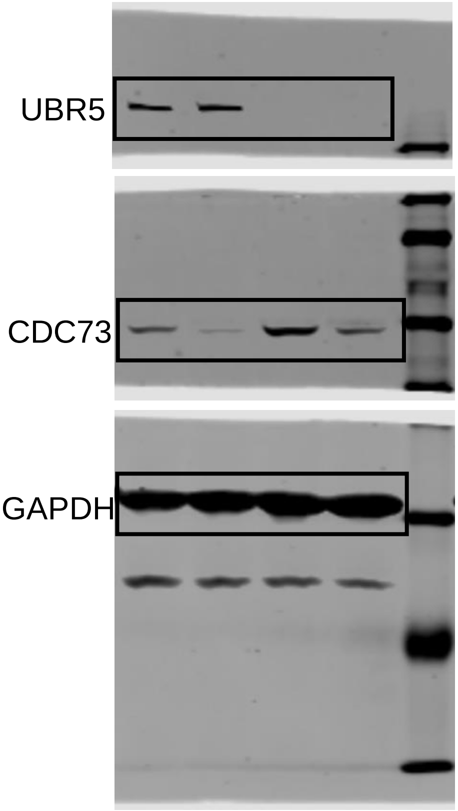

## Figure S7A

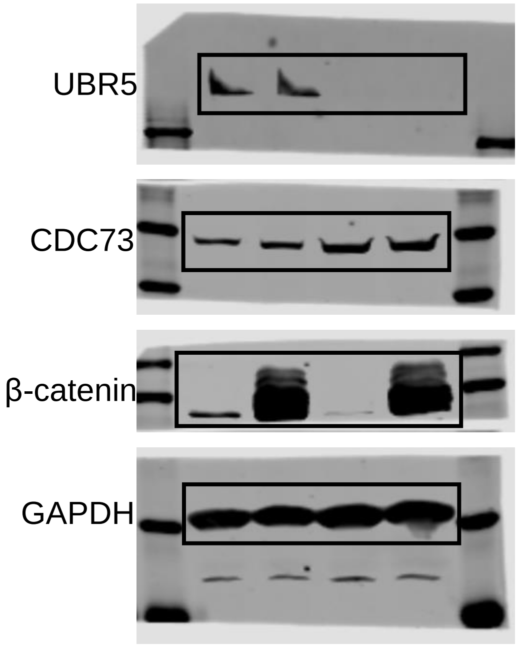


## Figure S7B

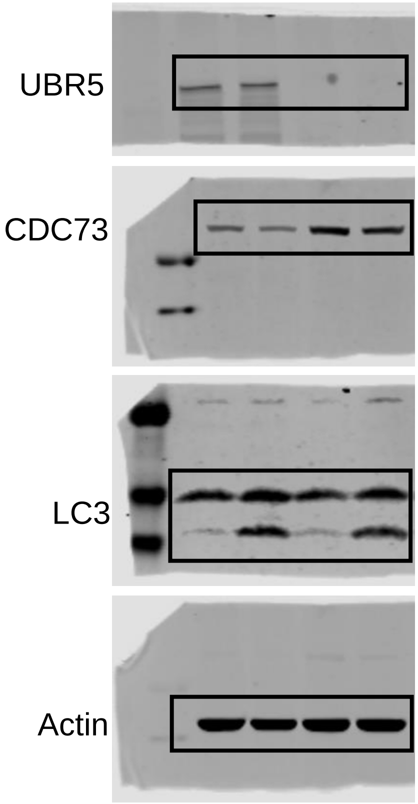


## Figure S7C

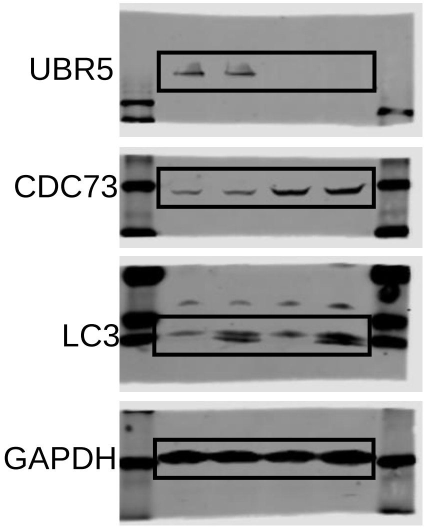

# Original immunofluorescence images

## Figure 5C

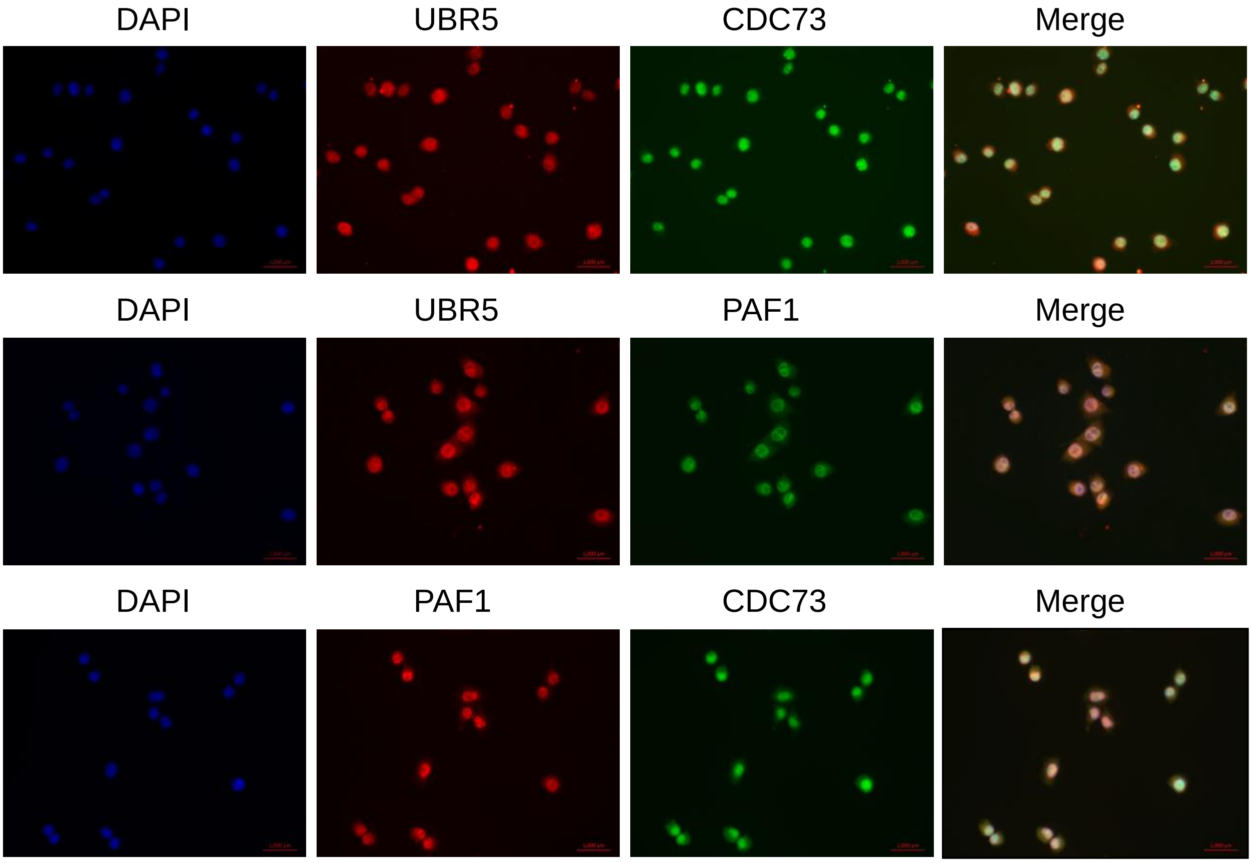

## Figure 3B

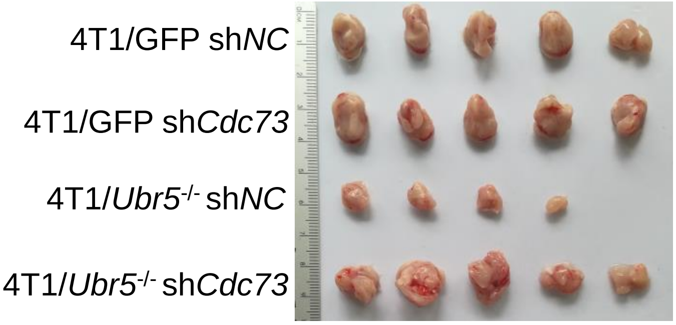


## Figure 4D

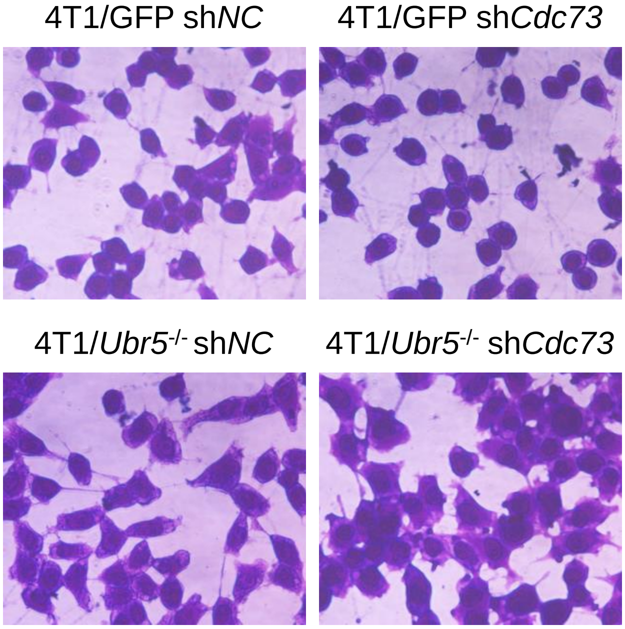


## Figure 3H

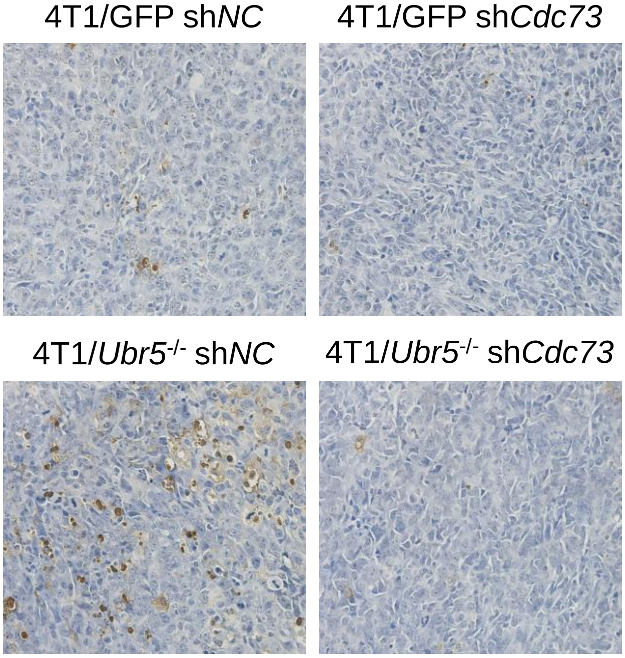


## Figure 4H

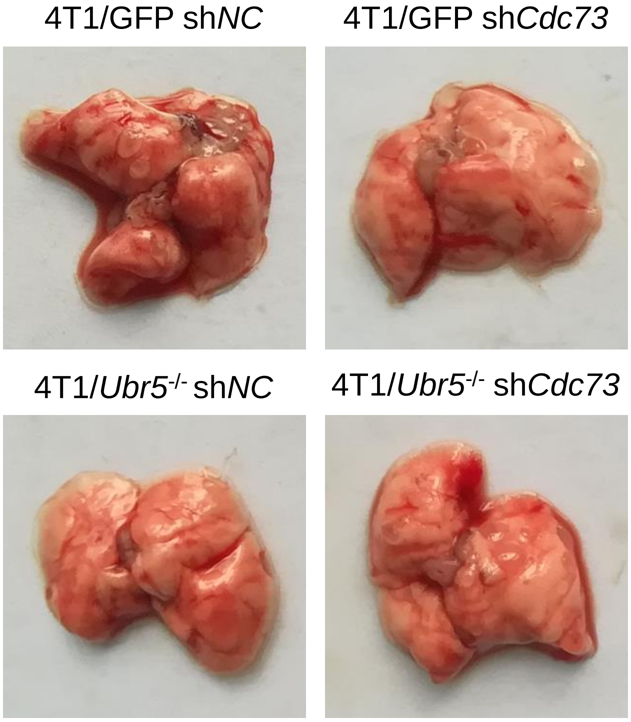


## Figure 4J

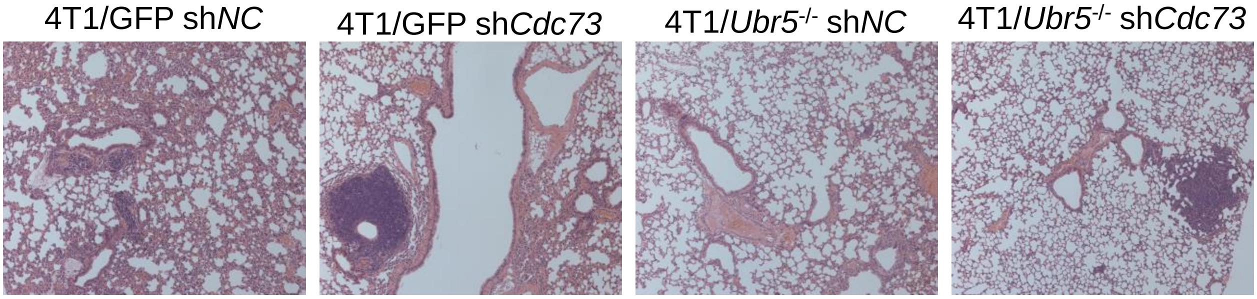

# Figure S5B

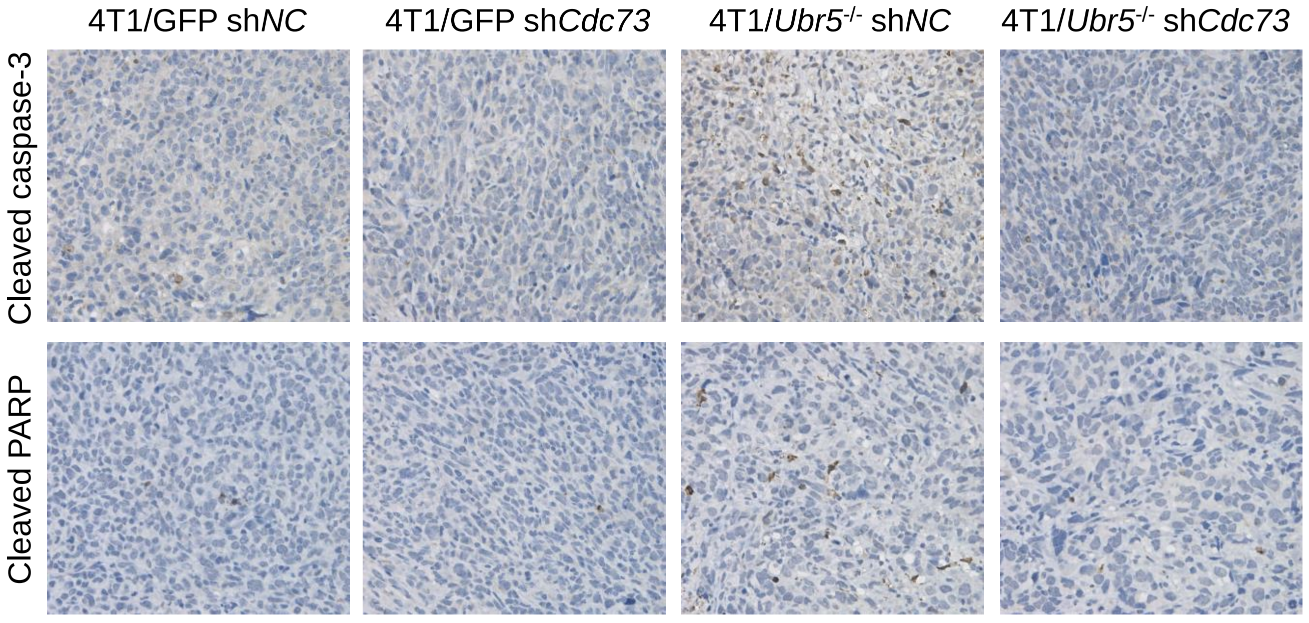

4T1/GFP shNC
4T1/GFP shCdc73
4T1/Ubr5-/- shNC
4T1/Ubr5-/- shCdc73
Cleaved caspase-3
Cleaved PARP
